# The Brain’s Topographical Organization Shapes Dynamic Interaction Patterns That Support Flexible Behavior Based on Rules and Long-Term Knowledge

**DOI:** 10.1523/JNEUROSCI.2223-23.2024

**Published:** 2024-03-25

**Authors:** Xiuyi Wang, Katya Krieger-Redwood, Baihan Lyu, Rebecca Lowndes, Guowei Wu, Nicholas E. Souter, Xiaokang Wang, Ru Kong, Golia Shafiei, Boris C. Bernhardt, Zaixu Cui, Jonathan Smallwood, Yi Du, Elizabeth Jefferies

**Affiliations:** ^1^CAS Key Laboratory of Behavioral Science, Institute of Psychology, Chinese Academy of Sciences, Beijing 100101, China; ^2^Department of Psychology, University of Chinese Academy of Sciences, Beijing 100049, China; ^3^Department of Psychology, University of York, Heslington, York YO10 5DD, United Kingdom; ^4^Department of Biomedical Engineering, University of California, Davis, California 95616; ^5^Centre for Sleep and Cognition (CSC) & Centre for Translational Magnetic Resonance Research (TMR), Yong Loo Lin School of Medicine, National University of Singapore, Singapore 117549, Singapore; ^6^Department of Psychiatry, Perelman School of Medicine, University of Pennsylvania, Philadelphia, Pennsylvania 19104; ^7^McConnell Brain Imaging Centre, Montreal Neurological Institute and Hospital, McGill University, Montreal, Quebec H3A 2B4, Canada; ^8^Chinese Institute for Brain Research, Beijing 102206, China; ^9^Department of Psychology, Queens University, Kingston, Ontario K7L 3N6, Canada; ^10^CAS Center for Excellence in Brain Science and Intelligence Technology, Shanghai 200031, China

**Keywords:** cortical topography, default mode network, dorsal attention network, flexible cognition, frontoparietal control network

## Abstract

Adaptive behavior relies both on specific rules that vary across situations and stable long-term knowledge gained from experience. The frontoparietal control network (FPCN) is implicated in the brain's ability to balance these different influences on action. Here, we investigate how the topographical organization of the cortex supports behavioral flexibility within the FPCN. Functional properties of this network might reflect its juxtaposition between the dorsal attention network (DAN) and the default mode network (DMN), two large-scale systems implicated in top-down attention and memory-guided cognition, respectively. Our study tests whether subnetworks of FPCN are topographically proximal to the DAN and the DMN, respectively, and how these topographical differences relate to functional differences: the proximity of each subnetwork is anticipated to play a pivotal role in generating distinct cognitive modes relevant to working memory and long-term memory. We show that FPCN subsystems share multiple anatomical and functional similarities with their neighboring systems (DAN and DMN) and that this topographical architecture supports distinct interaction patterns that give rise to different patterns of functional behavior. The FPCN acts as a unified system when long-term knowledge supports behavior but becomes segregated into discrete subsystems with different patterns of interaction when long-term memory is less relevant. In this way, our study suggests that the topographical organization of the FPCN and the connections it forms with distant regions of cortex are important influences on how this system supports flexible behavior.

## Significance Statement

Adaptive behavior depends on adjudicating between specific rules that vary across situations. The frontoparietal control network (FPCN) helps guide this process through its interactions with other brain regions. We examined how local topographical features support this function of the FPCN. Subnetworks within the FPCN share key anatomical and functional features with adjacent systems linked to external attention and long-term knowledge. This topographical architecture supports the emergence of distinct interaction patterns: FPCN subnetworks act cohesively when long-term memory can support behavior but segregate when long-term memory is not aligned with current goals. Our study shows that, in addition to dynamic interaction with spatially distant cortical regions, local topographical features of the FPCN play a significant role in flexible behavior.

## Introduction

**Figure 1.**
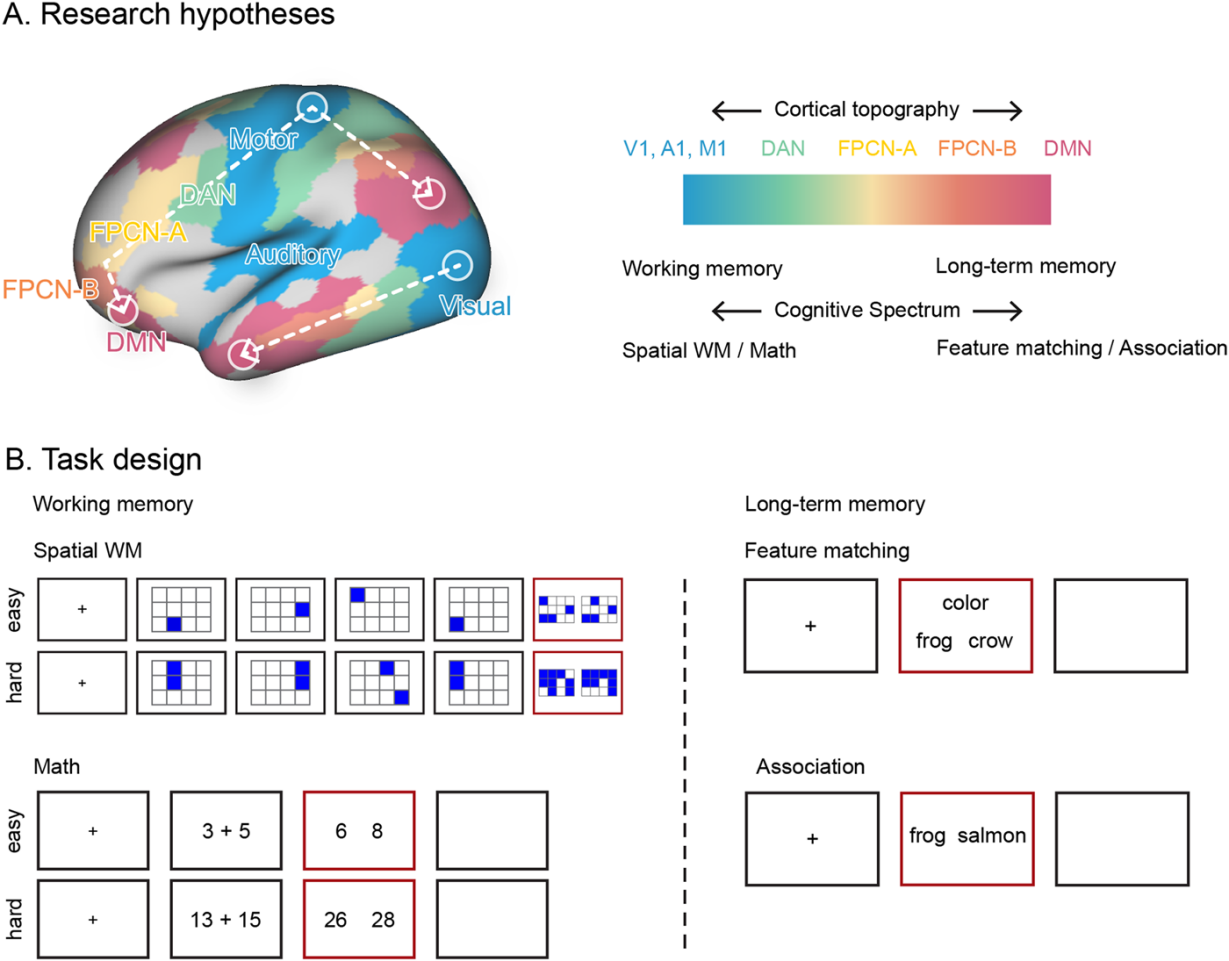
The research hypothesis and task design. ***A***, The research framework. Brain organization relates to cortical topography: brain regions supporting perceptual/motor features are maximally separated from heteromodal aspects of long-term memory, with control regions in the middle. This principal dimension of topographical organization might relate to a “cognitive spectrum” capturing distinctions between tasks that rely on recently presented information in working memory versus long-term knowledge (other spectrums may also exist). Regions of FPCN-A are closer to sensory-motor regions and more posterior than those of FPCN-B on the cortical mantle. These networks were identified using Kong et al.'s parcellation approach (Kong et al., 2021), which generates individual-specific parcellations with greater homogeneity. Other networks, such as DAN and DMN, are also divided into subnetworks. The subnetworks of visual, motor, DAN, and DMN are merged here for illustration. See Figure 3 for the distribution of each subnetwork. ***B***, Task design. To tap working memory, we included two tasks: a spatial working memory task required participants to keep track of sequentially presented locations, while math decisions involved maintaining and manipulating numbers which rely more on working memory. To tap long-term memory, we included two tasks that required controlled retrieval of knowledge; a semantic feature matching task required participants to match probe and target concepts according to a particular semantic feature (color or shape), while a semantic association task involved deciding if pairs of words were linked in meaning. Response periods are indicated by a red box. V1, visual; A1, auditory; M1, motor; DAN, dorsal attention network; FPCN, frontoparietal control network; DMN, default mode network. WM, working memory.

Human behavior is flexible, guided at times by information in working memory, which allows for temporary and effortful maintenance of relevant rules (Kok et al., 2016; Ekman et al., 2020), and at other times, by long-term memory, such as general knowledge of the world (Pandya et al., 2015). Situations that involve task-relevant rules rely on the brain's multiple demand system (Duncan, 2010), including dorsal attention network (DAN; Long and Kuhl, 2018) and frontoparietal control network (FPCN; Fedorenko et al., 2013). When cognition and behavior are supported by memory-based information, regions within the default mode network (DMN) play a pivotal role (Smallwood et al., 2021). However, recent perspectives focusing on the brain's topography suggest a more complex relationship between FPCN and DMN. For example, the principal mode of functional connectivity highlights similarity within these two networks (Margulies et al., 2016), and they exhibit analogous patterns in how functional signals change with space, since both networks show a rapid change in connectivity over small changes in cortical location (Leech et al., 2023). Consistent with these topographical perspectives, FPCN can be divided into two subnetworks: FPCN-A and FPCN-B (Fig. 1*A*; Yeo et al., 2011; Braga and Buckner, 2017; Dixon et al., 2018; Kam et al., 2019; Murphy et al., 2020; Yin et al., 2022). In a pioneering study, Dixon et al. (2018) revealed distinct activation and connectivity patterns for these subsystems, with FPCN-A more connected to DAN and FPCN-B more connected to DMN. Additionally, Murphy et al. (2020) demonstrated that interactions between FPCN subsystems and DMN can predict working memory performance. Leveraging this nascent body of evidence, our study addresses two questions: (1) how do underlying anatomical differences within FPCN subsystems contribute to their functional differentiation, and (2) how do these topographically separated systems adapt their interaction patterns to interface with sometimes anticorrelated networks (DAN and DMN), supporting flexible behavior (Dixon et al., 2018; Murphy et al., 2020; Yin et al., 2022; Godbersen et al., 2023).

We hypothesized that FPCN subnetworks show topographical differences that give rise to functional differences. FPCN-A is adjacent to DAN, while FPCN-B is more anterior on the cortical mantle and closer to DMN (Fig. 1*A*). This proximity of FPCN-A to DAN, and of FPCN-B to DMN, is anticipated to play a pivotal role in generating distinct cognitive modes relevant to working memory and long-term memory (Fig. 1). This proposal is related to evidence that cortical geometry (i.e., shape) influences function (Pang et al., 2023) and that the principal dimension describing functional differentiation within the cortex captures the physical sequence of networks on the cortical surface (Margulies et al., 2016), extending from sensory systems at one end to DMN at the other, with FPCN in between. A similar cortical hierarchy is observed across anatomy (Glasser et al., 2016; Huntenburg et al., 2017; Burt et al., 2018), correspondence between structure and function (Preti and Van De Ville, 2019; Vázquez-Rodríguez et al., 2019; Baum et al., 2020; Valk et al., 2022), and evolution (Hill et al., 2010; Xu et al., 2020; Sydnor et al., 2021). Although the broad topographical patterns are established (Mesulam, 1998; Buckner and Krienen, 2013), variations across these different measurements leave the exact locations of FPCN subsystems uncertain (Margulies et al., 2016; Burt et al., 2018; Raut et al., 2020). Therefore, we characterized the exact topographical positions of FPCN subsystems using multiple metrics.

Notably, while this topographical organization anticipates higher similarity for adjacent networks, the brain also produces flexible patterns of behavior based on task demands. By examining functional similarity within adjacent DMN, FPCN, and DAN across multiple tasks, we can establish that varying patterns of similarity are not solely a consequence of network adjacency. This clarifies how the topographical organization of the cortical mantle enables diverse interactions of FPCN with networks linked to top-down attention and long-term memory, yielding different landscapes of neural activity in response to situational demands.

## Materials and Methods

### Experimental design and statistical analysis

We investigated how the topography of FPCN subsystems relates to their functional interactions with other networks. The FPCN subsystems were defined using Kong et al.'s parcellation (Kong et al., 2021), which subdivides the FPCN into subnetworks that capture the inherent functional variability of the FPCN. This method enabled us to generate individual-specific parcellations with heightened homogeneity and to depict brain organization more accurately.

This study included three datasets: one open dataset—the Human Connectome Project (HCP)—and two task fMRI datasets collected at the University of York, United Kingdom. We analyzed the structural magnetic resonance imaging (MRI) and resting-state functional MRI (rsfMRI) data of 245 unrelated participants who completed all four resting-state scans from the S900 release of HCP dataset to investigate the cortical geometry, anatomical hierarchy, functional hierarchy, and functional connectivity patterns. We primarily employed paired *t* tests to characterize the topographical locations of FPCN subsystems.

We then employed multiple tasks to establish functional differentiation of the FPCN subsystems and to uncover how their interaction patterns change across contexts to support flexible behavior. By examining functional similarity across task states, we can establish how topography supports different cognitive modes, while also confirming that patterns of similarity are not solely a consequence of network adjacency. To distinguish control processes related to recently presented information in working memory from control processes for long-term memory, we examined two nonsemantic tasks requiring the maintenance of information in working memory, which have been analyzed previously (Wang et al., 2020, 2021, 2022), and two semantic long-term memory tasks scanned in York, United Kingdom. In the nonsemantic dataset, 31 participants completed easy and hard spatial working memory and arithmetic tasks originally designed to localize domain general control regions (Fedorenko et al., 2013). The math task falls in the working memory domain because it requires participants to continuously manipulate and update numerical information in their mind, a process that is integral to working memory function. This is particularly evident in the task's more challenging conditions, which demand significant mental computation and the retention of intermediate results for problem-solving. In the semantic dataset, 31 participants performed a semantic feature matching task in which participants were asked to match probe and target concepts (presented as written words) according to color or shape and a semantic association task in which they were asked to decide if pairs of words were semantically associated or not. The contrast between nonsemantic and semantic tasks allowed us to investigate how the interaction between control and other networks was modulated by task demands. We primarily employed paired *t* or independent tests, along with false discovery rate (FDR) corrections or family-wise error (FWE) corrections, to control for multiple comparisons.

### Participants

All participants were right-handed, native English speakers, with normal or corrected-to-normal vision, and no history of psychiatric or neurological illness. All participants provided informed consent. For the HCP dataset, the study was approved by the Institutional Review Board of Washington University at St. Louis. For the University of York dataset, the research was approved by the York Neuroimaging Centre and the Department of Psychology ethics committees.

The HCP sample involved data from 245 healthy volunteers (130 males, 115 females), aged 23–35 years (mean = 28.21; standard deviation (SD) = 3.67; Glasser et al., 2013).

Thirty-one healthy adults (26 females; age, mean ± SD = 20.60 ± 1.68; range, 18–25 years) performed the spatial working memory and math tasks. One participant with incomplete data was removed. A functional run was excluded if (1) the relative root mean square (RMS) framewise displacement (FD) was higher than 0.2 mm, (2) >15% of frames showed motion exceeding 0.25 mm, or (3) the accuracy of the behavior task was low (3 SD below the mean). If only one run of a task was left for a participant after exclusion, all their data for that task were removed. These exclusion criteria resulted in a final sample of 27 participants for both the spatial working memory task and the math task.

Thirty-one healthy adults performed the semantic long-term memory tasks (25 females; age, mean ± SD = 21.26 ± 2.93; range, 19–34 years). Using the same exclusion criteria above for the feature matching task, there were 23 participants with four runs, 4 participants with three runs, and 1 participant with two runs. For the association task, there were 24 participants with four runs, 3 participants with three runs, and 3 participants with two runs. An additional 30 native English speakers, who did not take part in the main functional MRI (fMRI) experiment, rated the color and shape similarity and semantic association strength for each word pair (21 females; age range, 18–24 years).

### Task paradigms

#### Spatial working memory task

Participants were required to maintain in memory four or eight sequentially presented locations in a 3 × 4 grid (Fedorenko et al., 2011), giving rise to easy and hard spatial working memory conditions. Stimuli were presented at the center of the screen across four steps. Each of these steps lasted for 1 s and highlighted one location on the grid in the easy condition and two locations in the hard condition. This was followed by a decision phase, which showed two grids side by side [i.e., two-alternative forced choice (2AFC) paradigm]. One grid contained the locations shown on the previous four steps, while the other contained one or two locations in the wrong place. Participants indicated their response via a button press and feedback was immediately provided within 2.5 s. Each run consisted of 12 experimental blocks (6 blocks per condition and 4 trials in a 32 s block) and 4 fixation blocks (each 16 s long), resulting in a total time of 448 s.

#### Math task

Participants were presented with an addition expression on the screen for 1.45 s and subsequently made a 2AFC decision, indicating their solution within 1 s. The easy condition used single-digit numbers, while the hard condition used two-digit numbers. Each trial ended with a blank screen lasting for 0.1 s. Each run consisted of 12 experimental blocks (with 4 trials per block) and 4 fixation blocks, resulting in a total time of 316 s.

#### Semantic feature matching task

Participants were required to make a yes/no decision, matching probe and target concepts (presented as words) according to a particular semantic feature (color or shape), specified at the top of the screen during each trial. The feature prompt, probe word, and target words were presented simultaneously. Half of the trials were matching trials in which participants would be expected to identify shared features, while the other half were non-matching trials in which participants would not be expected to identify shared features. For example, in a color matching trial, participants would answer “yes” to the word pair DALMATIANS–COWS, due to their color similarity, whereas they would answer “no” to COAL–TOOTH as they do not share a similar color.

We parametrically manipulated the degree of feature similarity between the probe and target concepts, using semantic feature similarity ratings taken from a separate group of 30 participants on a 5-point Likert scale. For instance, in color matching trials, the degree of color similarity between DALMATIANS and COWS was found to be very high (i.e., 4.8), while that between PUMA and LION was relatively low (i.e., 4.0), despite participants believing that the two trials had similar color. Conversely, in color non-matching trials, the degree of color similarity between CROW and HUMMINGBIRD was relatively high (i.e., 2.5), whereas that between COAL and TOOTH was very low (i.e., 1.2), even though participants perceived no similarity in color. For the matching trials, greater feature similarity facilitates the decision-making process, while for the non-matching trials, greater feature similarity makes the decision more difficult. This parametric design allowed us to model the effect of the difficulty of semantic decision-making in the neural data, and to test whether control subnetworks showed similar or opposite activation patterns.

This task included four runs and two conditions (two features, color and shape), presented in a mixed design. Each run consisted of four experimental blocks (two 2 min30 s blocks per feature), resulting in a total time of 10 min12 s. In each block, 20 trials were presented in a rapid event-related design. To maximize the statistical power of the rapid event-related fMRI data analysis, the stimuli were presented with temporal jitter randomized from trial to trial (Dale, 1999). The intertrial interval varied from 3 to 5 s. Each trial started with a fixation, followed by the feature, probe word, and target word presented centrally on the screen, triggering the onset of the decision-making period. The feature, probe word, and target word remained visible until the participant responded or for a maximum of 3 s. The condition order was counterbalanced across runs, and run order was counterbalanced across participants. Half of the participants pressed a button with their right index finger to indicate a matching trial and responded with their right middle finger to indicate a non-matching trial. The other half of the participants pressed the opposite buttons.

#### Semantic association task

Participants were asked to decide if pairs of words were semantically associated or not (i.e., yes/no decision as above) based on their own experience. Overall, there were roughly equal numbers of “related” and “unrelated” responses across participants. The same stimuli were used in both the semantic feature matching task and semantic association task. For example, DALMATIANS and COWS are semantically related; COAL and TOOTH are not. The feature and association tasks were separated by 1 week. Similarly, we parametrically manipulated the semantic association strength between the probe and target concepts, using semantic association strength ratings taken from a separate group of 30 participants on a 5-point Likert scale. For example, in related trials, the association strength between PUMA and LION is very strong, while for COWS and WHALE, it is relatively weak (although they are still both animals). In unrelated trials, the association strength between KINGFISHER and SCORPION is relatively high, while that between BANANA and BRICK is very low, although participants thought neither were related. For the related trials, stronger associations would facilitate decision-making, while for unrelated trials, stronger associations interfere with the decision-making. This parametric design allowed us to model the effect of the difficulty of semantic decision-making in the neural data and to test whether control subnetworks showed similar or opposite activation patterns.

This task included four runs, presented in a rapid event-related design. Each run consisted of 80 trials, with approximately half being related and half being unrelated trials. The procedure was the same as the feature matching task, except only two words were presented on the screen.

### Image acquisition

#### Image acquisition of HCP dataset

MRI acquisition protocols of the HCP dataset have been described (Barch et al., 2013; Glasser et al., 2013). Images were acquired using a customized 3 T Siemens Connectome scanner having a 100 mT/m SC72 gradient set and using a standard Siemens 32-channel radiofrequency receive head coil. Participants underwent the following scans: structural [at least one T1-weighted (T1w) magnetization-prepared rapid gradient echo (MPRAGE) and one 3D T2-weighted (T2w) sampling perfection with application optimized contrast (SPACE) scan at 0.7 mm isotropic resolution] and rsfMRI (4 runs × 14 min and 33 s). Since not all participants completed all scans, we only included 339 unrelated participants from the S900 release. Whole-brain rsfMRI and task fMRI data were acquired using identical multiband echoplanar imaging (EPI) sequence parameters of 2 mm isotropic resolution with a repetition time (TR) of 720 ms.

Subjects were considered for data exclusion based on the mean and mean absolute deviation of the relative RMS motion across either four rsfMRI scans or one diffusion MRI scan, resulting in four summary motion measures. If a subject exceeded 1.5 times the interquartile range (in the adverse direction) of the measurement distribution in two or more of these measures, the subject was excluded. In addition, functional runs were flagged for exclusion if >25% of frames exceeded 0.2 mm FD (FD_power). These above exclusion criteria were established before performing the analysis (Faskowitz et al., 2020; Sporns et al., 2021). The data of 91 participants were excluded because of excessive head motion and the data of another 3 participants were excluded because their resting data did not have all the time points. In total, the data of 245 participants were analyzed after exclusions.

#### Image acquisition of York nonsemantic dataset

MRI acquisition protocols have been described previously (Wang et al., 2020, 2021). Structural and functional data were collected on a Siemens Prisma 3 T MRI scanner at the York Neuroimaging Centre. The scanning protocols included a T1w MPRAGE sequence with whole-brain coverage. The structural scan used the following: acquisition matrix of 176 × 256 × 256 and voxel size 1 × 1 × 1 mm^3^; TR, 2,300 ms; and echo time (TE) = 2.26 ms. Functional data were acquired using an EPI sequence with an 80º flip angle and using generalized autocalibrating partially parallel acquisitions (GRAPPA) with an acceleration factor of 2 in 3 × 3 × 4 mm voxels in 64-axial slices. The functional scan used the following: 55 3-mm-thick slices acquired in an interleaved order (with 33% distance factor); TR, 3,000 ms; TE, 15 ms; and field of view (FOV), 192 mm.

#### Image acquisition of York semantic dataset

Whole-brain structural and functional MRI data were acquired using a 3 T Siemens MRI scanner utilizing a 64-channel head coil, tuned to 123 MHz at York Neuroimaging Centre, University of York. The functional runs were acquired using a multiband multiecho (MBME) EPI sequence, each 11.45 min long [TR, 1.5 s; TE, 12, 24.83, 37.66 ms; 48 interleaved slices per volume with slice thickness of 3 mm (no slice gap); FOV, 24 cm (resolution matrix = 3 × 3 × 3; 80 × 80); 75° flip angle; 455 volumes per run; 7/8 partial Fourier encoding and GRAPPA (acceleration factor, 3, 36 ref. lines); multiband acceleration factor, 2]. Structural T1w images were acquired using an MPRAGE sequence (TR, 2.3 s; TE, 2.3 s; voxel size, 1 × 1 × 1 isotropic; 176 slices; flip angle, 8°; FOV, 256 mm; interleaved slice ordering). We also collected a high-resolution T2w scan using an EPI sequence (TR, 3.2 s; TE, 56 ms; flip angle, 120°; 176 slices; voxel size, 1 × 1 × 1 isotropic; FOV, 256 mm).

### Image preprocessing

#### Image preprocessing of HCP dataset

We used HCP's minimal preprocessing pipelines (Glasser et al., 2013). Briefly, for each subject, structural images (T1w and T2w) were corrected for spatial distortions. FreeSurfer v5.3 was used for accurate extraction of cortical surfaces and segmentation of subcortical structures (Dale et al., 1999; Fischl et al., 1999). To align subcortical structures across subjects, structural images were registered using nonlinear volume registration to the Montreal Neurological Institute (MNI152) space. Functional images (rest and task) were corrected for spatial distortions, head motion, and mapped from volume to surface space using ribbon-constrained volume to surface mapping.

Subcortical data were also projected to the set of extracted subcortical structure voxels and combined with the surface data to form the standard Connectivity Informatics Technology Initiative (CIFTI) grayordinate space. Data were smoothed by a 2 mm full width at half maximum (FWHM) kernel in the grayordinates space. Rest data were additionally identically cleaned for spatially specific noise using spatial independent component analysis (ICA) + FMRIB’s ICA-based X-noiseiifier (FIX) (Salimi-Khorshidi et al., 2014) and global structured noise using temporal ICA (Glasser et al., 2018). For accurate cross-subject registration of cortical surfaces, a multimodal surface matching (MSM) algorithm (Robinson et al., 2014) was used to optimize the alignment of cortical areas based on features from different modalities. MSMSulc (“sulc”: cortical folds average convexity) was used to initialize MSMAll, which then utilized myelin, resting-state network, and rfMRI visuotopic maps. Myelin maps were computed using the ratio of T1w/T2w images (Salimi-Khorshidi et al., 2014). The HCP's minimally preprocessed data include cortical thickness maps (generated based on the standardized FreeSurfer pipeline with combined T1/T2 reconstruction). For this study, the standard-resolution cortical thickness maps (32,000 mesh) were used.

#### Image preprocessing of York non-semantic and semantic dataset

The York datasets were preprocessed using fMRIPrep 20.2.1 (Esteban et al., 2018; RRID:SCR_016216), which is based on Nipype 1.5.1 (Gorgolewski et al., 2011; RRID:SCR_002502). The details about preprocessing using fMRIPrep are provided in the subsequent subsections.

##### Anatomical data preprocessing

The T1w image was corrected for intensity nonuniformity with N4BiasFieldCorrection (Tustison et al., 2010), distributed with ANTs 2.3.3 (Avants et al., 2008; RRID:SCR_004757), and used as T1w reference throughout the workflow. The T1w reference was then skull-stripped with a Nipype implementation of the antsBrainExtraction.sh workflow (from ANTs), using OASIS30ANTs as target template. Brain tissue segmentation of the cerebrospinal fluid (CSF), white matter (WM), and gray matter (GM) was performed on the brain-extracted T1w using fast FSL 5.0.9 (Zhang et al., 2001; RRID:SCR_002823). Brain surfaces were reconstructed using recon-all from FreeSurfer 6.0.1 (Dale et al., 1999; RRID:SCR_001847), and the brain mask estimated previously was refined with a custom variation of the method to reconcile ANTs-derived and FreeSurfer-derived segmentations of the cortical gray matter of Mindboggle (Klein et al., 2017; RRID:SCR_002438). Volume-based spatial normalization to two standard spaces (MNI152NLin2009cAsym, MNI152NLin6Asym) was performed through nonlinear registration with antsRegistration (ANTs 2.3.3), using brain-extracted versions of both T1w reference and the T1w template. The following templates were selected for spatial normalization: ICBM 152 Nonlinear Asymmetrical template version 2009c (Fonov et al., 2009; RRID:SCR_008796; TemplateFlow ID: MNI152NLin2009cAsym) and FSL's MNI ICBM 152 nonlinear 6th Generation Asymmetric Average Brain Stereotaxic Registration Model (Evans et al., 2012; RRID:SCR_002823; TemplateFlow ID: MNI152NLin6Asym).

##### Functional data preprocessing

For each of the blood oxygen level dependent (BOLD) runs per subject, the following preprocessing was performed. First, a reference volume and its skull-stripped version were generated using a custom methodology of fMRIPrep. A B0-nonuniformity map (or fieldmap) was estimated based on a phase difference map calculated with a dual-echo gradient-recall echo sequence, processed with a custom workflow of SDCFlows inspired by the epidewarp.fsl script and further improvements in HCP Pipelines (Glasser et al., 2013). The fieldmap was then coregistered to the target EPI reference run and converted to a displacements field map (amenable to registration tools such as ANTs) with FSL's fugue and other SDCflows tools. Based on the estimated susceptibility distortion, a corrected EPI reference was calculated for a more accurate coregistration with the anatomical reference. The BOLD reference was then coregistered to the T1w reference using bbregister (FreeSurfer) which implements boundary-based registration (Greve and Fischl, 2009). Coregistration was configured with six degrees of freedom. Head motion parameters with respect to the BOLD reference (transformation matrices and six corresponding rotation and translation parameters) were estimated before any spatiotemporal filtering using mcflirt (FSL 5.0.9; Jenkinson et al., 2002). BOLD runs were slice time corrected using 3dTshift from AFNI 20160207 (SCR_005927). Since multiecho BOLD data was supplied in the York Semantic dataset, the tedana T2* workflow was used to generate an adaptive T2* map and optimally weighted combination of all supplied single echo time series. This optimally combined time series was then carried forward for all subsequent preprocessing steps. The BOLD time series were resampled onto the following surfaces (FreeSurfer reconstruction nomenclature): fsaverage. Grayordinate files (Glasser et al., 2013) containing 91,000 samples were also generated using the highest-resolution fsaverage as intermediate standardized surface space. Several confounding time series were calculated based on the preprocessed BOLD: FD, DVARS (the derivative of RMS variance over voxels), and three region-wise global signals. FD was computed using two formulations following previous work [absolute sum of relative motion (Power et al., 2014), relative RMS displacement between affines (Jenkinson et al., 2002)]. FD and DVARS were calculated for each functional run, both using their implementations in Nipype (Power et al., 2014). Three global signals were extracted within the CSF, the WM, and the whole-brain masks. The confound time series derived from head motion estimates and global signals were expanded with the inclusion of temporal derivatives and quadratic terms for each (Satterthwaite et al., 2013). Frames that exceeded a threshold of 0.5 mm FD or 1.5 standardized DVARS were annotated as motion outliers. All resamplings were performed with a single interpolation step by composing all the pertinent transformations (i.e., head motion transform matrices, susceptibility distortion correction when available, and coregistrations to anatomical and output spaces). Gridded (volumetric) resamplings were performed using antsApplyTransforms (ANTs), configured with Lanczos interpolation to minimize the smoothing effects of other kernels (Lanczos, 1964). Nongridded (surface) resamplings were performed using mri_vol2surf (FreeSurfer). fMRIPrep used Nilearn 0.6.2 (Abraham et al., 2014; RRID:SCR_001362), mostly within the functional processing workflow. The resulting data were in CIFTI 64,000-vertex grayordinate space. The left hemisphere had 29,696 vertices and right hemisphere had 29,716 vertices in total after removing the medial wall.

Postprocessing of the outputs of fMRIPrep version 20.2.1 (Esteban et al., 2018) was performed using the eXtensible Connectivity Pipeline (XCP; Satterthwaite et al., 2013; Ciric et al., 2018). For each CIFTI run per subject, the following postprocessing was performed: before nuisance regression and filtering any volumes with FD >0.3 mm (Satterthwaite et al., 2013; Power et al., 2014) were flagged as outliers and excluded from nuisance regression. In total, 36 nuisance regressors were selected from the nuisance confound matrices of fMRIPrep output. These nuisance regressors included six motion parameters, global signal, mean white matter, and mean CSF signal with their temporal derivatives, and the quadratic expansion of six motion parameters, tissue signals, and their temporal derivatives (Satterthwaite et al., 2013; Ciric et al., 2017, 2018). These nuisance variables were accounted for in the BOLD data using linear regression—as implemented in Scikit-Learn 0.24.2 (Pedregosa et al., 2011). Residual time series from this regression were then bandpass filtered to retain signals within the 0.01−0.08 Hz frequency band. The processed BOLD was smoothed using Connectome Workbench with a Gaussian kernel size of 6.0 mm (FWHM). Processed functional time series were extracted from residual BOLD using Connectome Workbench (Glasser et al., 2013). Many internal operations of XCP use Nibabel (Abraham et al., 2014), numpy (Harris et al., 2020), and scipy (Harris et al., 2020).

### Structural and task fMRI analysis

#### Individual-specific parcellation

Considering the anatomical and functional variability across individuals (Mueller et al., 2013; Laumann et al., 2015; Braga and Buckner, 2017; Gordon et al., 2017), we estimated individual-specific areal-level parcellation using a multisession hierarchical Bayesian model (MS-HBM; Kong et al., 2019, 2021). To estimate individual-specific parcellation, we acquired “pseudo-resting state” time series, in which the task activation model was regressed from spatial working memory, math, feature matching, and semantic association fMRI data (Fair et al., 2007) using xcp_d (https://github.com/PennLINC/xcp_d). The task activation model and nuisance matrix were regressed out using AFNI's 3dTproject (for similar implementation, see Cui et al., 2020).

Using a group atlas, this method calculates intersubject resting-state functional connectivity variability and intrasubject resting-state functional connectivity variability and finally parcellates each single subject based on this prior information. As in Kong et al. (2019, 2021), we used MS-HBM to define 400 individualized parcels belonging to 17 discrete individualized networks for each participant, where the FPCN was divided into 3 subnetworks. This allowed us to explore the heterogeneity of FPCN. Specifically, we calculated all participants' connectivity profiles, created the group parcellation using the average connectivity profile of all participants, estimated the intersubject and intrasubject connectivity variability, and finally calculated each participant's individualized parcellation. This parcellation imposed the Markov random field spatial prior. We used a well-known areal-level parcellation approach, that is, the local gradient approach (gMS-HBM), which detects local abrupt changes (i.e., gradients) in resting-state functional connectivity across the cortex (Cohen et al., 2008). A previous study (Schaefer et al., 2018) has suggested combining local gradient (Cohen et al., 2008; Gordon et al., 2016) and global clustering (Yeo et al., 2011) approaches for estimating areal-level parcellations. Therefore, we complemented the spatial contiguity prior in contiguous MS-HBM (cMS-HBM) with a prior based on local gradients in resting-state functional connectivity. This encouraged adjacent brain locations with gentle changes in functional connectivity to be grouped into the same parcel. We used the pair of parameters (i.e., β value = 50; *w* = 30; and *c* = 30), which was optimized using our own dataset. The same parameters were also used in Kong et al. (2021). Vertices were parcellated into 400 cortical regions (200 per hemisphere). To parcellate each of these parcels, we calculated the average time series of enclosed vertices to improve the signal-to-noise ratio using Connectome Workbench software. This parcel-based time series was used for all the subsequent analyses. The same method and parameters were used to generate the individual-specific parcellation for participants in the HCP dataset using the resting-state time series except that the task regression was not performed.

##### Homogeneity of parcels

To evaluate whether a functional parcellation is successful, parcel homogeneity is commonly used (Gordon et al., 2016; Kong et al., 2019, 2021). Parcel homogeneity was calculated as the average Pearson's correlations between fMRI time courses of all pairs of vertices within each parcel, adjusted for parcel size and summed across parcels (Schaefer et al., 2018; Kong et al., 2019, 2021). Higher homogeneity means that vertices within the same parcel share more similar time courses and indicates better parcellation quality. To summarize the parcel homogeneity, we averaged the homogeneity value across parcels. We calculated the parcel homogeneity for each run of each participant for each task using the individual-specific parcellation and then averaged them across runs for each participant for each task. We also calculated the parcel homogeneity using the canonical Yeo 17-network group atlas. Using the resting-state data of the HCP dataset, Kong et al. (2021) demonstrated that homogeneity within MS-HBM-based individualized parcels was greater than that in the canonical Yeo 17-network group atlas, which does not consider variation in functional neuroanatomy. A similar pattern was observed using York Nonsemantic and Semantic datasets (Wang et al., 2023). We also observed that the homogeneity of the semantic tasks in the York Semantic dataset was higher than that of the nonsemantic tasks in the York Nonsemantic dataset (Wang et al., 2023). The potential reason might be that we collected T2w images for the semantic tasks to improve skull-stripping, resulting in a better outcome for pial surface reconstruction. Given the known heterogeneity within the FPCN, as well as within the DAN and DMN, we did not merge any subnetworks as was done by Dixon et al. (2018) and Murphy et al. (2020).

#### Cortical geometry—global minimum distance to primary sensory-motor landmarks

To reveal how physical proximity to structural landmarks corresponding to primary sensory-motor regions influences the function of regions, we calculated the geodesic distance between each parcel and key landmarks associated with the primary visual, auditory, and somatomotor cortex. These values were used to identify the minimum geodesic distance to primary sensory-motor regions for each parcel. Three topographical landmarks were used: the central sulcus corresponding to the primary somatosensory/motor cortex; temporal transverse sulcus indicating primary auditory cortex; and calcarine sulcus, marking the location of primary visual cortex. Since the cortical folding patterns vary across participants, and the individual variability in cortical folding increases with cortical surface area (Van Essen et al., 2019), both the shapes of these landmarks and the number of vertices within each landmark might show individual differences. We used participant-specific landmark label files to locate the participant-specific vertices belonging to each landmark and participant-specific parcellation to locate the vertices within each parcel.

Geodesic distance along the “midthickness” of the cortical surface (halfway between the pial and white matter) was calculated using the Connectome Workbench software with an algorithm that measures the shortest path between two vertices on a triangular surface mesh (Mitchell et al., 1987; O’Rourke, 1999). This method returns distance values independent of mesh density. Geodesic distance was extracted from surface geometry (GIFTI) files, following surface-based registration (Robinson et al., 2014). To ensure that the shortest paths would only pass through the cortex, vertices representing the medial wall were removed from the triangular mesh for this analysis.

We calculated the minimum geodesic distance between each vertex and each landmark. Specifically, for the central sulcus, we calculated the geodesic distance between vertex *i* outside the central sulcus and each vertex within it (defined for each individual). We then identified vertex *j* within the central sulcus closest to vertex *i* and extracted this value as the minimum geodesic distance for vertex *i* to this landmark. To compute the minimum geodesic distance for parcel *k* to the central sulcus, we averaged the minimum distance across all the grayordinate vertices in parcel *k* to the vertices within the central sulcus. The same procedure was applied to calculate minimum geodesic distance between each parcel and all three sensory-motor landmarks (central sulcus, temporal transverse sulci, and calcarine sulcus). From these three minimum geodesic distances, we selected the lowest distance value (i.e., the closest landmark to parcel *k*) as the global minimum distance to sensory-motor regions for parcel *k*. We then averaged the mean minimum distance of all parcels within each network for each participant and then sorted the networks by the mean minimum distance across participants. Finally, we examined whether mean minimum distance of FPCN-A and FPCN-B were different by performing a paired *t* test.

#### Anatomical hierarchy—myelin content and cortical thickness

We measured myelin, which is a noninvasive and valid proxy for anatomical hierarchy, and captures this hierarchy more effectively than cortical thickness (Burt et al., 2018). Gray matter myelin content can be measured via the cortical T1w/T2w map, a structural neuroimaging map defined by the contrast ratio of T1- to T2-weighted (T1w/T2w) magnetic resonance images (Glasser and Van Essen, 2011; Glasser et al., 2013, 2016; Salimi-Khorshidi et al., 2014).

Human T1w/T2w maps were obtained from the HCP in the surface-based CIFTI file format. To generate these maps, high-resolution T1- and T2-weighted images were first registered to a standard reference space using a state-of-the-art areal-feature-based technique (Glasser and Van Essen, 2011; Glasser et al., 2013, 2016).

Each participant has a myelin map, with each vertex having a myelin value (i.e., T1w/T2w ratio). We calculated the myelin value of each parcel by averaging the myelin values of all vertices within the parcel for each participant, using the individual-specific parcellation. Similarly, we calculated the myelin value of each network by averaging the myelin values of all parcels within each network for each participant and then sorted the networks by the mean myelin value across participants. Finally, we examined whether the myelin values of the FPCN-A and FPCN-B differed by performing paired *t* tests.

Cortical thickness, which coarsely tracks changes in cytoarchitecture and myelin content, can be seen as a pragmatic surrogate for cortical microstructure. Therefore, we also examined cortical thickness of each network which measures the width of gray matter across networks. The procedure was similar to that for myelin mapping, except we used the cortical thickness maps and extracted the cortical thickness of each vertex for each participant.

#### Function hierarchy—principal connectivity gradient analysis

To examine the relative position of networks on the principal gradient axis of intrinsic connectivity, we performed dimension reduction analysis on the resting-state functional connectivity matrix of the HCP dataset. First, we calculated the resting-state functional connectivity for each run of each participant using the method mentioned below, Constructing fMRI functional connectivity matrices. We then averaged these individual connectivity matrices to generate a group-averaged connectivity matrix. We used the BrainSpace Toolbox (Vos de Wael et al., 2020) to extract 10 group-level gradients from the group-averaged connectivity matrix (dimension reduction technique, diffusion embedding; kernel, none; sparsity, 0.9), following the methodology of previous studies (Mckeown et al., 2020; Wang et al., 2020). Using identical parameters, gradients were then calculated for each individual using their average 400 × 400 resting-state functional connectivity matrix across four runs. These individual-level gradient maps were aligned to the group-level gradient maps using Procrustes rotation, facilitating the comparison between the group- and individual-level gradients (*N* iterations = 10). This analysis produced 10 group-level gradients and 10 individual-level gradients for each participant, explaining maximal whole-brain connectivity variance in descending order. For 238 out of 245 participants, the first gradient, which explained the maximal variance, was the principal gradient which captures the separation between unimodal and transmodal regions (Margulies et al., 2016). Next, we averaged the first gradient values of all parcels within each network for each participant for whom the first gradient was the principal gradient and then sorted the networks by the mean gradient values across participants. Finally, we examined whether the gradient values of FPCN-A and FPCN-B differed by performing paired *t* tests.

#### Comparing the evolutionary expansion and cross-species similarity between FPCN-A and FPCN-B

We used the evolutionary expansion map and cross-species similarity map provided by Xu et al. (2020), available at https://github.com/TingsterX/alignment_macaque-human. The surface areal expansion map was calculated as the ratio of the human area to the macaque area at each corresponding vertex on human and macaque surfaces (Xu et al., 2020). Cross-species similarity was calculated by comparing the whole-brain patterns of functional connectivity in macaques and humans (Xu et al., 2020). We extracted the value of each vertex and calculated the mean value for each parcel within the group parcellation. Finally, we compared the values between FPCN-A and FPCN-B by conducting independent *t* tests.

#### Feature extraction of the time-series data

We used the highly comparative time-series analysis toolbox, hctsa (Fulcher et al., 2013; Fulcher and Jones, 2017), to extract massive features from the time-series data. Using a time-series dataset, hctsa allowed us to transform each time series into a set of over 7,700 features, with each feature encoding a different scientific analysis method (Fulcher et al., 2013; Fulcher and Jones, 2017). The extracted features include, but are not limited to, distributional properties, entropy and variability, autocorrelation, time-delay embeddings, and nonlinear properties of a given time series (Fulcher et al., 2013; Fulcher, 2018). The hctsa feature extraction analysis was performed on the parcellated fMRI time series for each participant, each task, and each run separately. Following this procedure, we removed the outputs of the operations that produced errors and normalized the remaining features (∼6,900 features) across parcels using an outlier-robust sigmoidal transform. The normalized feature matrix (400 parcels × ∼7,000 features × 4 runs) was used for subsequent classification analysis and feature similarity analysis.

#### Classification analysis

To reveal network similarity in a data-driven approach, we performed classification analysis using the normalized feature matrix. This analysis was motivated by the observation that specific brain regions possess distinct features, equipping them to support different functions, while regions with similar features are likely to support similar functions, irrespective of whether they exhibit positive or negative functional connectivity. For instance, early visual areas and DMN are situated at opposing ends of the timescale hierarchy. The former is characterized by the shortest timescale, marked by rapid temporal autocorrelation decay, while the latter has the longest, characterized by gradual autocorrelation decay (Honey et al., 2012; Simony et al., 2016; Blank and Fedorenko, 2020; Ito et al., 2020; Raut et al., 2020). Consequently, neural representations in early visual areas are minimally influenced by prior knowledge, whereas DMN regions are significantly swayed by it (González-García et al., 2018). In addition, regions with more similar features often support parallel functions. For example, while the two large-scale networks in the transmodal regions, FPCN and DMN, typically exhibit negative functional connectivity, they share attributes like long timescales, indicating they can process inputs over extended periods. Thus, their neural representations are influenced by prior knowledge (González-García et al., 2018) and goal states maintained over time (Wang et al., 2021). These observations suggest that feature similarity can provide valuable information about the functional similarity of networks beyond functional connectivity. Furthermore, features can vary depending on tasks and brain states (Golesorkhi et al., 2021; Wolff et al., 2022), indicating their potential to reflect variations in network interaction across tasks.

##### Balanced accuracy of classification analysis

To examine whether we could accurately classify the network labels of each parcel using the extracted features, we performed a classification task to determine the accuracy with which a classifier can learn a mapping from time-series features of parcels to their network labels. We combined the normalized features from all runs of each task for each participant and performed multiclass classification (17 networks labels) using scikit-learn (Pedregosa et al., 2011), a machine learning library written in Python.

For multiclass classification, we trained linear support vector machine classifiers (sklearn.svm.SVC) to find the hyperplane that maximally separates the samples belonging to different classes. As the number of parcels in networks varies (e.g., both FPCN-A and FPCN-B have 25 parcels, while FPCN-C has 23), we reported balanced classification accuracy to account for the imbalance of observations across networks (Brodersen et al., 2010; Kelleher et al., 2015). Specifically, balanced accuracy was calculated as the arithmetic mean of sensitivity (i.e., true positive rate, which measures the proportion of correctly predicted positives) and specificity (i.e., true negative rate, which measures the proportion of correctly identified negatives). To prevent overfitting and optimistic performance estimates, we performed fivefold cross-validation employing a within-subject cross-validation approach. We merged data from all runs per task per participant, creating datasets with 1,600 samples (400 parcels × 4 runs) for semantic tasks and 800 samples (400 parcels × 2 runs) for nonsemantic tasks. This dataset underwent a fivefold cross-validation, with data from the same and different runs used in training and testing phases. To access whether the classification accuracy was significantly above chance level for each participant and each task, we performed permutation-based multiclass classification analysis by randomly shuffling network labels 1,000 times within all runs for each participant for each task. This established an empirical distribution of classification accuracy scores under the null hypothesis where there is no association between features and network labels (Ojala and Garriga, 2010). We recognize that using data from both same and different runs during training and testing may bias accuracy, stemming from data nonindependence. However, this nonindependence is present both in actual testing and when establishing an empirical distribution of classification accuracy through permutation-based multiclass classification. This implies that the nonindependence potentially biases both actual and permuted accuracies similarly. Therefore, comparing actual accuracy with the empirical distribution remains a valid approach and does not undermine our conclusions.

Given the high dimensionality of the data used for classification (∼7,000 features and 1,600 samples for semantic tasks; and 800 samples for nonsemantic tasks), machine learning with many more features than samples are challenging. We addressed the challenge posed by the curse of dimensionality, which describes the increase in noise and redundancy with the explosive nature of increasing data dimensions (Hughes, 1968). To explore the curse of dimensionality, we performed classification using all the features and subsets of features, ranging from 500 to 7,000 features, with increments of 500 features. Subsets of features were the top features that make major contributions in the classification when including all the features. We observed minimal accuracy cost with fewer features and no curse of dimensionality. Specifically, the accuracy increased with the number of features, peaking at 4,000 features, beyond which a slight decrease was noted but remained above chance level. Consequently, we used 4,000 features for the classification accuracy and confusion matrix presented in the main text.

##### Confusion matrix of classification analysis

While the classification accuracy allows us to check if we can correctly classify the network labels of parcels, it alone might not reveal the detailed information needed to diagnose the performance of our model. For instance, in multiclass classification, high classification accuracy could be due to all classes being predicted equally well or because one or two classes are being disproportionately favored by the model. Therefore, to gain a deeper understanding of our classification model's performance, we analyzed the confusion matrix, which summarizes prediction results. In this matrix, each row represents an instance of the actual class (i.e., an actual network), and each column represents an instance of the predicted class (i.e., the predicted network). The diagonal elements indicate the number of points where the predicted label matches the true label, while off-diagonal elements represent mislabeled instances by the classifier. Higher values on the diagonal correspond to a greater number of correct predictions. The confusion matrix elucidates how our classification model performs when making predictions, providing insights into not only the errors made by the classifier but, more importantly, the types of errors that occur. Specifically, it allows us to explore network similarity by analyzing the classification output and how this similarity varies with the task.

Due to the imbalance of observations across networks, we normalized the confusion matrices by the number of elements in each class. In this study, we focused on reporting the normalized confusion matrix for the subnetworks of FPCN. We investigated whether the percentages of predicted networks were significantly different by conducting paired *t* tests. We applied FDR at *p* = 0.05 to control for multiple comparisons.

#### Constructing feature similarity matrices

To investigate how subnetworks of FPCN interact with DAN and DMN, we calculated both task and resting-state feature similarity. For each normalized feature matrix, we calculated Pearson’s correlation coefficients and transformed them to Fisher *z* values to represent the pairwise feature similarity between the time-series features of all possible combinations of brain parcels. This process resulted in a 400 × 400 feature similarity matrix for each individual and each run, representing the strength of the similarity in the local temporal fingerprints of brain areas. Finally, we averaged the estimates of feature similarity both within networks and between pairs of networks to construct a network-by-network feature similarity matrix. The same method was used to calculate the resting-state feature similarity using the HCP dataset and construct a network-by-network feature similarity matrix.

#### Constructing fMRI redundancy matrices

To investigate how subnetworks of FPCN interact with DAN and DMN, we employed the concept of time-delayed mutual information (TDMI), a specialized version for analyzing temporal dependencies in time-series data. TDMI extends mutual information by introducing time delays, assessing the mutual information between a variable's current state and its past states with a time lag. This extension enables us to understand how past information predicts the current state over time. To calculate redundancy, we utilized the recently developed integrated information decomposition approach, as introduced by Mediano et al. (2021) and Luppi et al. (2022). Integrated information decomposition expands on the partial information decomposition theory, breaking down TDMI into redundant (information provided by both sources), unique (by one source but not the other), and synergistic information (jointly by their combination) concerning both past and present states of the involved regions. We specifically focused on temporally persistent redundancy, aiming to quantify the extent to which distinct brain regions redundantly carry information about the brain's future trajectory, serving as an indicator of functional interaction. This measure was calculated using the Gaussian solver implemented in the JIDT toolbox, based on their hemodynamic response function-deconvolved BOLD signal time series. We calculated the redundant interaction for each pair of brain regions, resulting in a 400 × 400 redundancy matrix for each participant, per task and per run. Finally, we averaged the estimates of redundancy within networks, and between pairs of networks, to construct a network-by-network redundancy matrix, similar to the one constructed from the feature similarity data.

While the organization of redundancy shares similarities with traditional functional connectivity, as pairs of regions with more correlated time courses are more likely to provide redundant information, they are not identical. This is evident by the moderate correlation between the functional connectivity matrix and the redundancy matrix (mean = 0.62; SD = 0.24; Luppi et al., 2022). Notably, different ensembles of regions can transiently shift from being redundancy dominated to synergy dominated (Varley et al., 2023). This shift suggests that two regions may have strong functional connectivity while not consistently sharing information throughout the entire observation period. This distinction between measures also indicates that persistent redundancy offers valuable insights into the dynamics of information sharing between brain regions that are not fully captured by functional connectivity.

#### Constructing fMRI functional connectivity matrices

To investigate how FPCN subnetworks interact with DAN and DMN, we further calculated both task and resting-state functional connectivity. We opted not to use the traditional psychophysiological interaction method for measuring task-state functional connectivity due to its potential to inflate activation-induced task-state functional connectivity, which may identify regions that are active rather than interacting during the task (Cole et al., 2019). Since task activations can spuriously inflate task-based functional connectivity estimates, it is necessary to correct for task-timing confounds by removing the first-order effect of task-evoked activations (i.e., mean evoked task-related activity, likely active during the task) prior to estimating task-state functional connectivity (likely interacting during the task; Cole et al., 2019). Specifically, we fitted the task timing for each task using a finite impulse response model, a method that has been widely used and shown to reduce both false positives and false negatives in functional connectivity estimation (Norman-Haignere et al., 2012; Cocuzza et al., 2020; McCormick et al., 2022). In semantic tasks, approximately five time points were modeled for each trial. For the nonsemantic task, roughly 2.5 time points were modeled for each trial.

Following task regression, we demeaned the residual time series for each parcel and quantified the functional connectivity using Pearson’s correlation for each participant, task, and run. Pearson’s correlation coefficients might be inflated due to the temporal autocorrelation in task fMRI time-series data (Allefeld et al., 2008). To address this, we corrected Pearson’s correlation using a novel correction approach, xDF, which accounts for both autocorrelation within each time series as well as instantaneous and lagged cross-correlations between the time series (Afyouni et al., 2019). This method provides an effective degrees of freedom estimator that addresses cross-correlations, thereby preventing inflation of Pearson’s correlation coefficients. Our goal was not to remove temporal autocorrelation but to enhance the precision and reliability of correlation estimates, reducing false functional connectivity between regions. We calculated xDF-adjusted *z*-scored correlation coefficients to assess the interregional relationships in BOLD time series, resulting in a 400 × 400 functional connectivity matrix for each participant, task, and run. Finally, we averaged these functional connectivity estimates within networks, and between pairs of networks, to construct a network-by-network functional connectivity matrix. The same method was used to calculate the resting-state functional connectivity of the HCP dataset and construct a corresponding network-by-network functional connectivity matrix, except without the task regression step.

#### Comparing feature similarity, redundancy, and functional connectivity difference between networks

To investigate whether FPCN-A showed greater feature similarity with DAN than FPCN-B did in each task, we calculated the average feature similarity between FPCN-A and DAN, and the feature similarity between FPCN-B and DAN, respectively, across all runs per participant per task, and then conducted paired *t* tests to compare the levels of similarity between these network pairs for each task. Similarly, we examined the feature similarity between FPCN-B and DMN versus FPCN-A and DMN. We conducted FDR correction at *p* = 0.05 to control for multiple comparisons. We investigate whether FPCN-A showed stronger redundancy and functional connectivity with DAN than FPCN-B did. We also examined whether FPCN-B showed greater redundancy and functional connectivity with DMN than FPCN-A did. The procedure was as above, except we extracted the redundancy and functional connectivity matrix, respectively.

We further investigated whether the task context influenced the feature similarity difference between network pairs using the maximum/minimum permutation test. We might expect that feature similarity between FPCN-A and DAN-A versus FPCN-A and FPCN-B would be greater in spatial working memory task than that in the association task because FPCN-A might shift more toward DAN in the spatial working memory task that requires an external goal but no memory retrieval. To examine this possibility, we calculated the mean feature similarity difference between FPCN-A and DAN-A versus FPCN-A and FPCN-B for each task and calculated the mean feature similarity difference between each task pair. To test for statistical significance, we permuted the task label 10,000 times; we then calculated the mean feature similarity difference between these two tasks to build a null distribution for each task pair. Since we included multiple task pairs, we used the permutation-based maximum mean feature similarity difference and minimum mean feature similarity difference values in the null distribution for each task pair to control the FWE rate (*p* = 0.05, FWE corrected). To evaluate significance, if the observed mean difference value was positive, we counted the percentage of times that mean difference values in the maximum null distribution were greater than the observed “true” mean difference values; in contrast, if the observed mean difference value was negative, we counted the percentage of times of mean difference values in the minimum null distribution were less than the observed “true” mean difference values.

Similarly, we investigated whether the task influenced the redundancy and functional connectivity difference between network pairs. The procedure was as above, except we extracted the redundancy and functional connectivity from the network-by-network redundancy and functional connectivity matrix, respectively. We conducted FDR correction at *p* = 0.05 to control for multiple comparisons.

#### Task fMRI univariate analysis

To reveal the functional differentiation between FPCN-A and FPCN-B, we examined whether they showed similar or opposite activation patterns. We identified regions that were activated or deactivated in the tasks by building a general linear model. We also examined regions where neural responses were modulated by task difficulty. For semantic tasks, we included one task mean regressor and one demeaned parametric regressor of semantic rating. We examined how the neural responses were negatively modulated by feature similarity rating in the matching trials and positively modulated in the nonmatching trials. For the semantic association task, we examined how neural responses were negatively modulated by semantic association strength rating in related trials and positively modulated in nonrelated trials. For the nonsemantic tasks, we included two regressors—easy and hard conditions—to reveal the regions showing greater activation in the hard than easy conditions. We also modeled incorrect trials as regressors of no interest in all tasks.

We extracted the β value for each parcel in these task conditions and tested whether they were significantly activated (i.e., above zero) or deactivated (i.e., below zero) relative to implicit baseline (i.e., fixation period) to explore the task mean effect. Then we considered parcels that showed stronger or weaker activation in the hard than that in the easy condition in the spatial working memory and math tasks and that the activations were positively modulated by semantic difficulty. These parcels were thought to support general executive control. Fixed-effects analyses were conducted using nilearn (Abraham et al., 2014) to estimate average effects across runs within each subject for each parcel. Then we conducted one-sample *t* tests to assess whether the estimated effect size (i.e., contrast) was significantly different from zero across all subjects. We applied FDR correction at *p* = 0.05 to control for multiple comparisons. Finally, we identified the network to which each parcel belonged according to Kong et al. (2021).

### Data and code accessibility

The HCP data is publicly available (https://www.humanconnectome.org/). The data collected at the University of York is not currently available due to insufficient consent. Researchers wishing to access the data should contact the Chair of the Research Ethics Committee of the York Neuroimaging Centre. Data will be released when this is possible under the terms of the UK general data protection regulation. Analysis code for this study is available at https://github.com/Xiuyi-Wang/Control_Project.

## Results

The results are divided into three sections: (1) first, we take an existing individualized parcellation of the cortex, identifying two large-scale distributed control networks, corresponding to FPCN-A and FPCN-B, and establish that parcels of these networks have reliably different topographical locations by quantifying their distance from sensory-motor regions using multiple metrics; (2) next, we ask how these differences in the topography of FPCN-A and FPCN-B relate to their functional interaction patterns; and (3) finally, we demonstrate how these interaction patterns produce flexible behavior across different task contexts.

We selected Kong et al.'s parcellation (Kong et al., 2021) as the most appropriate choice, since this parcellation subdivides the FPCN into subnetworks, which is essential given the inherent heterogeneity of the FPCN and because this method enables us to generate individual-specific parcellations with heightened homogeneity. The naming of FPCN-A and FPCN-B here was consistent with the original Yeo et al.'s parcellation (Yeo et al., 2011) and Kong et al.'s individualized parcellation (Kong et al., 2021), adapted from the Yeo et al.'s parcellation. However, the opposite naming has been used in some previous studies that investigate the functional differentiation of the FPCN (Dixon et al., 2018; Murphy et al., 2020). We did not use other parcellations, including those by Power et al. (2011), Gordon et al. (2016), and Glasser et al. (2016), because the FPCN was identified as a functional unit without further subdivisions in these atlases.

### The topographical characteristics of FPCN-A and FPCN-B

To establish whether the FPCN-A subnetwork is closer to sensory-motor systems while FPCN-B is proximal to the DMN, we examined the topographical positions of FPCN-A and FPCN-B on multiple metrics, including cortical geometry, anatomical characteristics, principal connectivity gradient values, cortical expansion, and cross-species functional similarity. Although these characteristics generally show similar topographical patterns—spanning from unimodal to transmodal (Mesulam, 1998; Buckner and Krienen, 2013; Margulies et al., 2016; Smallwood et al., 2021)—they do not perfectly align with each other. For example, the correspondence between structure and function is weaker in transmodal cortex (Vázquez-Rodríguez et al., 2019), and minimum physical distance to sensory-motor landmarks shows only a moderate correlation with the principal connectivity gradient (Margulies et al., 2016). This motivated us to comprehensively capture the topographical positioning of FPCN subnetworks across multiple metrics.

#### FPCN-A is physically closer to sensorimotor cortex than FPCN-B

**Figure 2.**
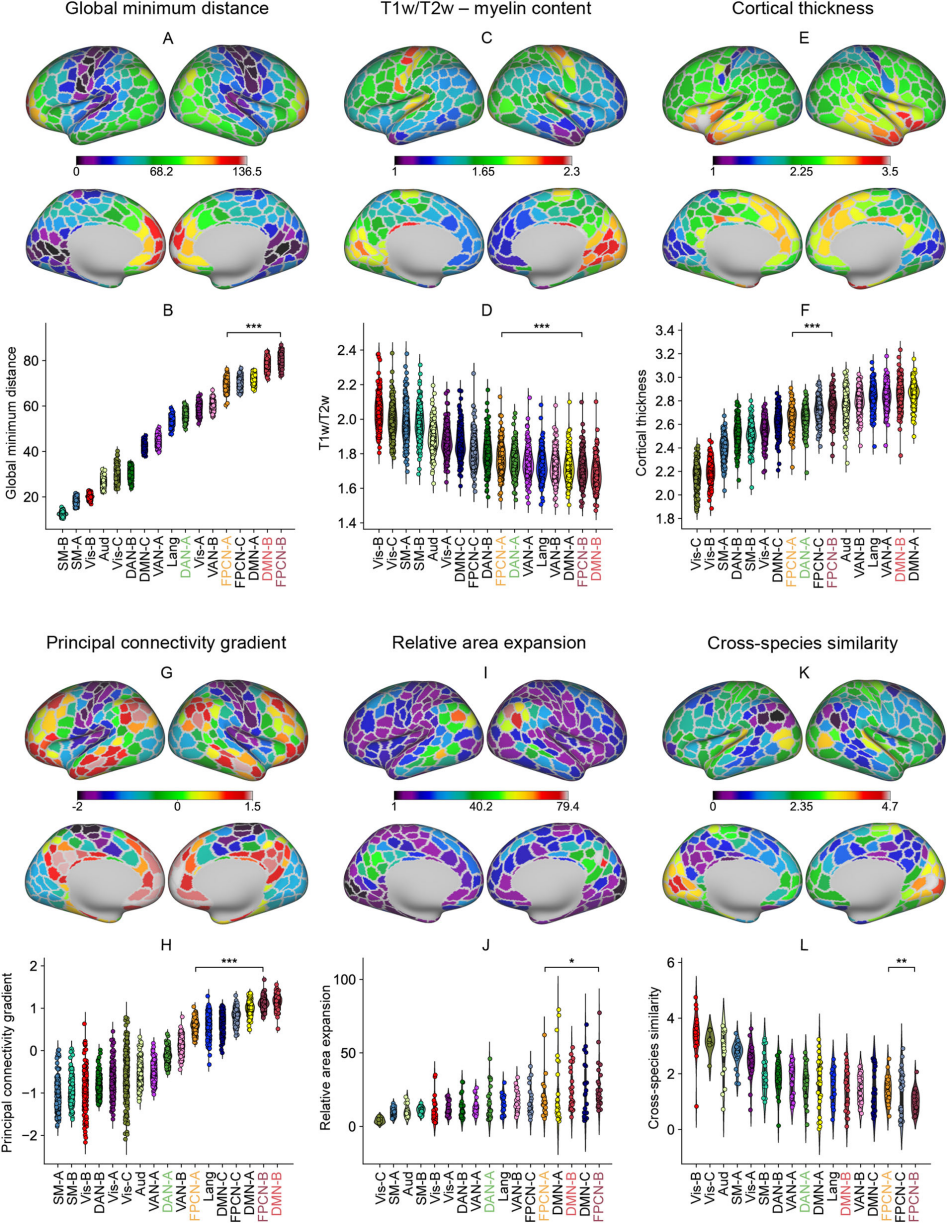
FPCN-A was closer to DAN and sensory-motor systems, while FPCN-B was proximal to DMN in physical distance, myelin content, cortical thickness, principal connectivity gradient values, cortical expansion, and cross-species in functional similarity. Each data point in ***B***, ***D***, ***F***, and ***H*** represents the data of one participant, whereas each data point in ***J*** and ***L*** represents the data of one parcel. We sorted the networks by their mean values across participants in ***B***, ***D***, ***F***, and ***H*** and across parcels in ***J*** and ***L***. ***A***, ***B***, The global minimum geodesic distance between each parcel and its closet sensory-motor landmark and the global minimum distance for each network (averaged across parcels). FPCN-A was closer to sensory-motor landmarks than FPCN-B. ***C***, ***D***, T1w/T2w values were significantly lower in association networks than those in sensory networks. The T1w/T2w value in FPCN-A was higher than that in FPCN-B and was closer to the value for sensory-motor cortex. ***E***, ***F***, The cortex was generally thicker in FPCN and DMN than that in sensory-motor networks. The cortex in FPCN-A was thinner than that in FPCN-B. ***G***, ***H***, The principal connectivity gradient that explained the most variance in resting-state fMRI captured the separation between sensory-motor and transmodal regions. FPCN-A was closer to the sensory-motor end of this gradient axis than FPCN-B. ***I***, ***J***, Sensory cortices expanded the least from the macaque to the human, while transmodal cortex expanded the most. FPCN-A showed less cortical expansion than FPCN-B. ***K***, ***L***, Regions of sensory-motor networks showed greater cross-species similarity between humans and macaques, whereas transmodal regions showed greater differences. FPCN-A showed greater cross-species similarity than FPCN-B. Vis, visual; Aud, auditory; SM, sensory-motor; DAN, dorsal attention network; VAN, ventral attention network; FPCN, frontoparietal control network; Lang, language; DMN, default mode network.

Distance from sensorimotor regions is thought to provide an organizing principle of functional differentiation within the cortex (Mesulam, 1998; Buckner and Krienen, 2013; Smallwood et al., 2021; Paquola et al., 2022). Therefore, our first analysis confirmed that there were systematic differences in the location of the FPCN-A and FPCN-B networks on the cortical surface, in terms of their physical distance to primary sensory-motor landmarks, using the HCP dataset. We calculated the geodesic distance between each parcel and three key landmarks associated with primary visual, auditory, and somatomotor cortices to identify the global minimum geodesic distance to primary sensorimotor regions for each parcel. Figure 2*A* shows a group-level representation of global minimum distance from sensory-motor cortex: transmodal regions are further from these landmarks. FPCN-A showed greater physical proximity to sensorimotor cortex than FPCN-B (*t*_(244)_ = −100.57; *p* < 0.001; paired *t* test; Fig. 2*A*,*B*).

#### FPCN-A was closer than FPCN-B to the unimodal end of the anatomical organization defined by myelin content and cortical thickness

Having confirmed topographical differences in the control subnetworks, we next examined whether these are mirrored in anatomical differences. The cortical T1w/T2w map—sensitive to regional variation in gray matter myelin content (Glasser and Van Essen, 2011; Glasser et al., 2016; Burt et al., 2018)—is thought to reflect an anatomical hierarchy, with sensorimotor regions showing greater myelination (Burt et al., 2018). We hypothesized that FPCN-A would have higher levels of myelination than FPCN-B since the analysis above showed that FPCN-A was closer to sensorimotor cortex. We analyzed participants' individual T1w/T2w maps and cortical thickness maps from the HCP dataset. As expected, myelin values were high in sensorimotor cortices and low in association cortices. Among the transmodal networks, FPCN-A had higher myelin values than FPCN-B (*t*_(244)_ = 46.89; *p* < 0.001; paired *t* test; Fig. 2*C*,*D*). In parallel, since cortical thickness coarsely tracks changes in cytoarchitecture and myelin content, we found that the cortex was generally thicker in heteromodal control networks and the DMN than in sensory-motor networks (Fig. 2*E*,*F*). FPCN-A had lower cortical thickness than FPCN-B (*t*_(244)_ = 34.08; *p* < 0.001; paired *t* test).

#### FPCN-A was closer to the unimodal end of the principal connectivity gradient than FPCN-B

Having established that anatomical differences reflect topographical proximity, we next explored whether resting-state functional connectivity shows a similar pattern. Global minimum distance is positively correlated with location on the principal connectivity gradient, which organizes neural systems along a spectrum from unimodal to transmodal cortex (Margulies et al., 2016; Wang et al., 2022). We therefore asked whether the parcels of FPCN-A were closer than those of FPCN-B to the unimodal end of the principal connectivity gradient. Dimension reduction analysis was performed on the HCP resting-state functional connectivity matrix. For 238 out of 245 participants, the dimension explaining the most variance corresponded to the principal gradient as described by Margulies et al. (2016): sensory-motor regions fell at one end of this dimension of connectivity (Fig. 2*G*, purple–blue), while transmodal areas were located at the other end (Fig. 2*H*, red–orange). We averaged the principal gradient values of all the parcels within each network for all participants for whom the principal gradient explained the most variance. We found that sensory-motor networks fell at one end, while control networks and the DMN were located at the other end. FPCN-A had lower values on the principal gradient than FPCN-B (*t*_(244)_ = 51.93; *p* < 0.001; paired *t* test; Fig. 2*G*,*H*), indicating that FPCN-A was closer to sensorimotor systems on this dimension of connectivity, while FPCN-B was closer to the DMN apex of the principal gradient.

#### FPCN-A showed less cortical expansion from macaque to human and showed greater similarity across species relative to FPCN-B

A prominent theory of cortical organization suggests that transmodal networks became untethered from sensorimotor systems through evolution (Buckner and Krienen, 2013). Therefore, we hypothesized that FPCN-B would show more cortical expansion and less cross-species similarity in functional connectivity than FPCN-A, since it is further from sensorimotor systems. We used the evolutionary expansion and cross-species similarity maps provided by Xu et al. (2020). To estimate surface areal expansion, the human surface area was divided by the macaque surface area at each corresponding vertex, and then the averages of all vertices within each parcel were calculated. We found that FPCN-B showed more expansion (*t*_(244)_ = 2.125; *p* = 0.039; paired *t* test; Fig. 2*I*,*J*) and less cross-species functional similarity than FPCN-A (*t*_(244)_ = −3.333; *p* = 0.002; paired *t* test; Fig. 2*K*,*L*).

### Functional interaction and activation patterns of FPCN-A and FPCN-B

FPCN-A and FPCN-B occupy distinct topographical positions: FPCN-A is closer to sensory-motor landmarks and DAN, while FPCN-B is closer to DMN in geodesic distance, anatomical features relating to myelination, functional connectivity patterns, and evolutionary markers. Since DAN and DMN sometimes show negative functional connectivity (Fox et al., 2005; Anderson et al., 2011; Chen et al., 2017; Dixon et al., 2017; Craig et al., 2018), FPCN subnetworks that are proximal to these systems may show a degree of functional separation that reflects their topography. To investigate this possibility, we analyzed multiple metrics of functional similarity, comparing DAN and DMN with FPCN subnetworks A and B to establish whether they showed dissociable patterns of functional recruitment in rest, working memory, and long-term memory tasks. We would expect greater functional similarity between FPCN-A and adjacent DAN, and between FPCN-B and adjacent DMN, if topography constrains brain function (Mesulam, 1998; Margulies et al., 2016; Buckner and DiNicola, 2019; Buckner and Margulies, 2019; Smallwood et al., 2021). We investigated this prediction in three complementary analyses examining (1) feature classification across networks, (2) functional coupling, and (3) activation and deactivation patterns modulated by task difficulty. Since different methods can reveal different facets of network interaction (Mediano et al., 2021; Luppi et al., 2022; Santoro et al., 2023; Varley et al., 2023), comprehensive evidence from various methods, contextualized within our broader understanding of brain anatomy and function, will provide the most reliable insights.

#### FPCN-A was more likely to be misclassified as DAN-A and FPCN-B was more likely to be misclassified as DMN-B

**Figure 3.**
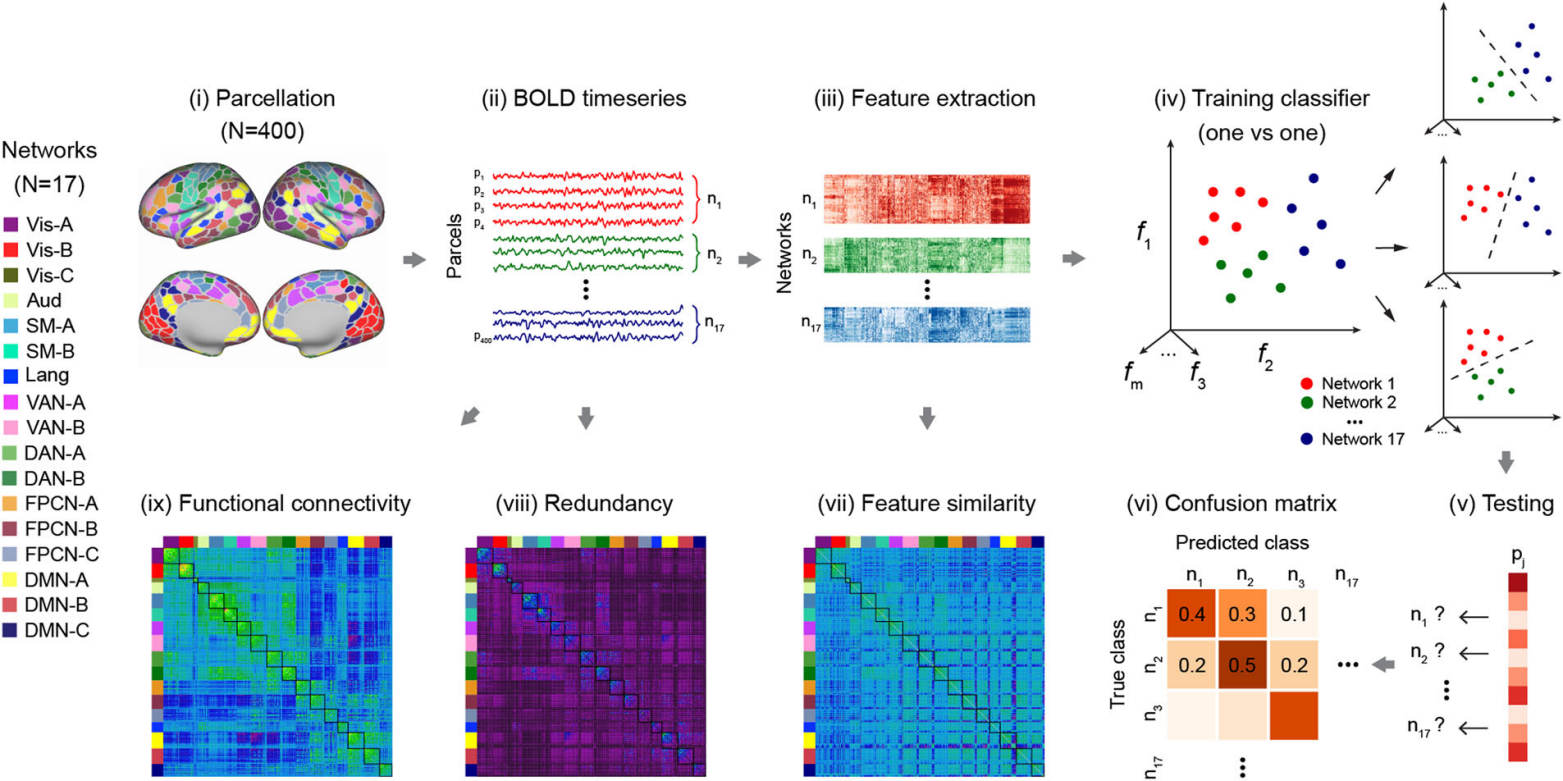
Overview of analytic approaches to study the functional interaction patterns of brain networks. ***i***, Individual-specific parcellation divided the whole brain into 400 parcels across 17 networks (Kong et al., 2021). ***ii***, Average time series of each parcel. ***iii***, Extraction of features of time series for each parcel. ***iv***, Multiclass classification analysis involved training a classifier to learn the mapping from time-series features of parcels to their network labels and ***v***, then to predict network labels for parcels. ***vi***, Network similarity was characterized by the normalized confusion matrix: networks with more similar functions would be more likely to be incorrectly classified as each other. ***vii***, Pearson’s correlation coefficients of the extracted features represent the pairwise feature similarity between all possible combinations of brain parcels. ***viii***, Redundancy quantifies how much information about the brain's future trajectory is redundantly predicted by distinct brain regions. We focused on temporally persistent redundancy, which corresponds to redundant information in the past of both regions that is also present in the future (Luppi et al., 2022). ***ix***, Functional connectivity involved calculating Pearson’s correlation coefficients between the time series of parcels. Vis, visual; Aud, auditory; SM, sensory-motor; DAN, dorsal attention network; VAN, ventral attention network; FPCN, frontoparietal control network; Lang, language; DMN, default mode network; WM, working memory.

To reveal network similarity, multiclass classification was used to predict the network labels of parcels using extracted features of the time-series data. These features include temporal autocorrelation, kurtosis, and entropy (Shafiei et al., 2020), among others, which may capture meaningful differences between different types of time series and thus represent promising candidates as quantitative phenotypes for distinguishing data of different types (see Materials and Methods, Feature extraction of the time-series data and Classification analysis; Fig. 3 for the analysis pipeline). This analysis was motivated by the observation that certain brain regions possess distinct features, which equip them to support different functions, while regions with similar features are suited to support similar functions, irrespective of whether they exhibit positive or negative functional connectivity. For instance, early visual areas and the DMN are situated at opposing ends of the timescale hierarchy. The former boasts the shortest timescale, marked by rapid temporal autocorrelation decay, while the latter has the longest, characterized by gradual autocorrelation decay (Honey et al., 2012; Simony et al., 2016; Blank and Fedorenko, 2020; Ito et al., 2020; Raut et al., 2020). Consistently, neural representations in early visual areas are minimally influenced by prior knowledge, whereas DMN regions are significantly swayed by it (González-García et al., 2018). In addition, regions with more similar features tend to support parallel functions. For example, while FPCN and DMN generally exhibit negative functional connectivity, they share some similar attributes including long timescales, suggesting they can process inputs over longer periods. As a result, their neural representations are shaped by prior knowledge (González-García et al., 2018) and goal states maintained over time (Wang et al., 2021). These observations suggest that feature similarity can provide valuable information about the functional similarity of networks beyond functional connectivity. This unbiased, hypothesis-free analysis that combines machine learning with feature extraction can objectively pinpoint the specific subnetworks within DAN and DMN that demonstrate a heightened functional similarity to FPCN subnetworks and elucidate the functions of FPCN subnetworks.

**Figure 4.**
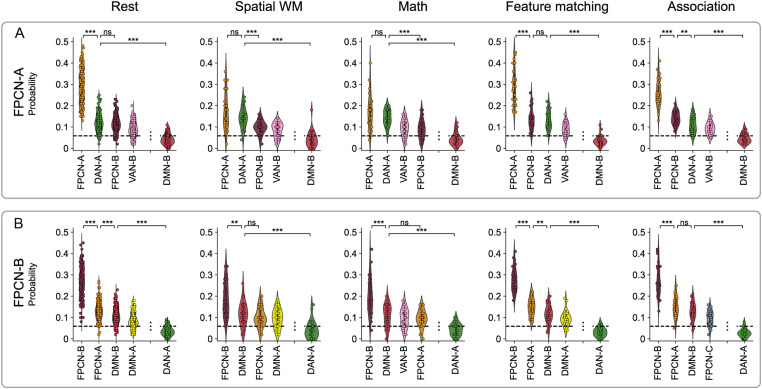
The probabilities of classifying FPCN-A and FPCN-B as each network (figure shows top four networks plus an additional comparator network). ***A***, FPCN-A was most likely to be incorrectly classified as DAN-A at rest and in nonsemantic tasks and as FPCN-B in semantic tasks. It was also misclassified as VAN-B but rarely misclassified as DMN-B. ***B***, FPCN-B was most likely to be misclassified as DMN-B in nonsemantic tasks and as FPCN-A in semantic tasks and at rest. It was also misclassified as DMN-A and but rarely misclassified as DAN-A. Dashed lines represent the chance level. (**p = 0.01; ***p = 0.001; ns = not significant, *p* > 0.05; FDR corrected).

We tested the hypothesis that parcels of FPCN-A and FPCN-B would be misclassified as belonging to different networks, reflecting their closest neighbors on the topographical spectrum (Ito et al., 2020; Raut et al., 2020; Shafiei et al., 2020). Specifically, the classifier might misclassify parcels of FPCN-A as belonging to DAN and FPCN-B as belonging to DMN. We found that classification accuracy was significantly greater than chance for each participant on each task (rest: mean = 0.37, SD = 0.04; spatial working memory: mean = 0.24, SD = 0.03; math: mean = 0.21, SD = 0.04; semantic feature matching: mean = 0.38, SD = 0.03; semantic association: mean = 0.37, SD = 0.03). Figure 4 shows the top four networks with the highest prediction probabilities within the normalized confusion matrix, plus an additional comparator network; the probabilities for other networks were similar to chance level.

By analyzing the classification output (i.e., the confusion matrix), we found that FPCN-A and FPCN-B showed similarity to different networks. Specifically, FPCN-A was most likely to be misclassified as DAN-A and FPCN-B, while FPCN-B was most likely to be misclassified as FPCN-A and DMN-B (Fig. 4). When the target network was FPCN-A, there was a higher probability that parcels would be misclassified as DAN-A than as DMN-B across rest and all the tasks (*p* < 0.05, FDR corrected; Fig. 4, Table 1). Conversely, when the target network was FPCN-B, parcels were more likely to be misclassified as DMN-B than as DAN-A (*p* < 0.05, FDR corrected; Fig. 4, Table 1). These results indicate that the time-series characteristics of FPCN-A were more similar to those of DAN-A and those of FPCN-B were more similar to those of DMN-B. Therefore, subsequent analyses were focused on the functional similarity between DAN-A, DMN-B, and FPCN subnetworks.

**Table 1.** FPCN-A was more likely to be misclassified as DAN-A than as DMN-B, while FPCN-B showed the opposite pattern

True network	Task	Mean probability		DAN-A vs DMN-B		
		DAN-A	DMN-B	*t*	*p*	*q*
FPCN-A	Rest	0.118	0.040	27.313	3.66 × 10^−76^	1.83 × 10^−75^
	Spatial WM	0.144	0.043	9.191	8.41 × 10^−10^	1.05 × 10^−09^
	Math	0.143	0.044	9.887	1.81 × 10^−10^	3.02 × 10^−10^
	Feature matching	0.132	0.036	10.908	5.82 × 10^−12^	1.45 × 10^−11^
	Association	0.111	0.047	7.087	7.01 × 10^−8^	1.31 × 10^−7^
FPCN-B	Rest	0.036	0.111	−26.184	8.00 × 10^−73^	4.00 × 10^−72^
	Spatial WM	0.042	0.116	−5.070	2.52 × 10^−5^	4.20 × 10^−5^
	Math	0.044	0.111	−5.516	7.64 × 10^−6^	9.55 × 10^−6^
	Feature matching	0.032	0.115	−10.358	2.00 × 10^−11^	3.34 × 10^−11^
	Association	0.027	0.128	−14.129	8.50 × 10^−15^	2.12 × 10^−14^

*q* is the FDR-corrected *p* value.

#### FPCN-A showed greater functional interaction with DAN-A, while FPCN-B showed greater interaction with DMN-B

**Figure 5.**
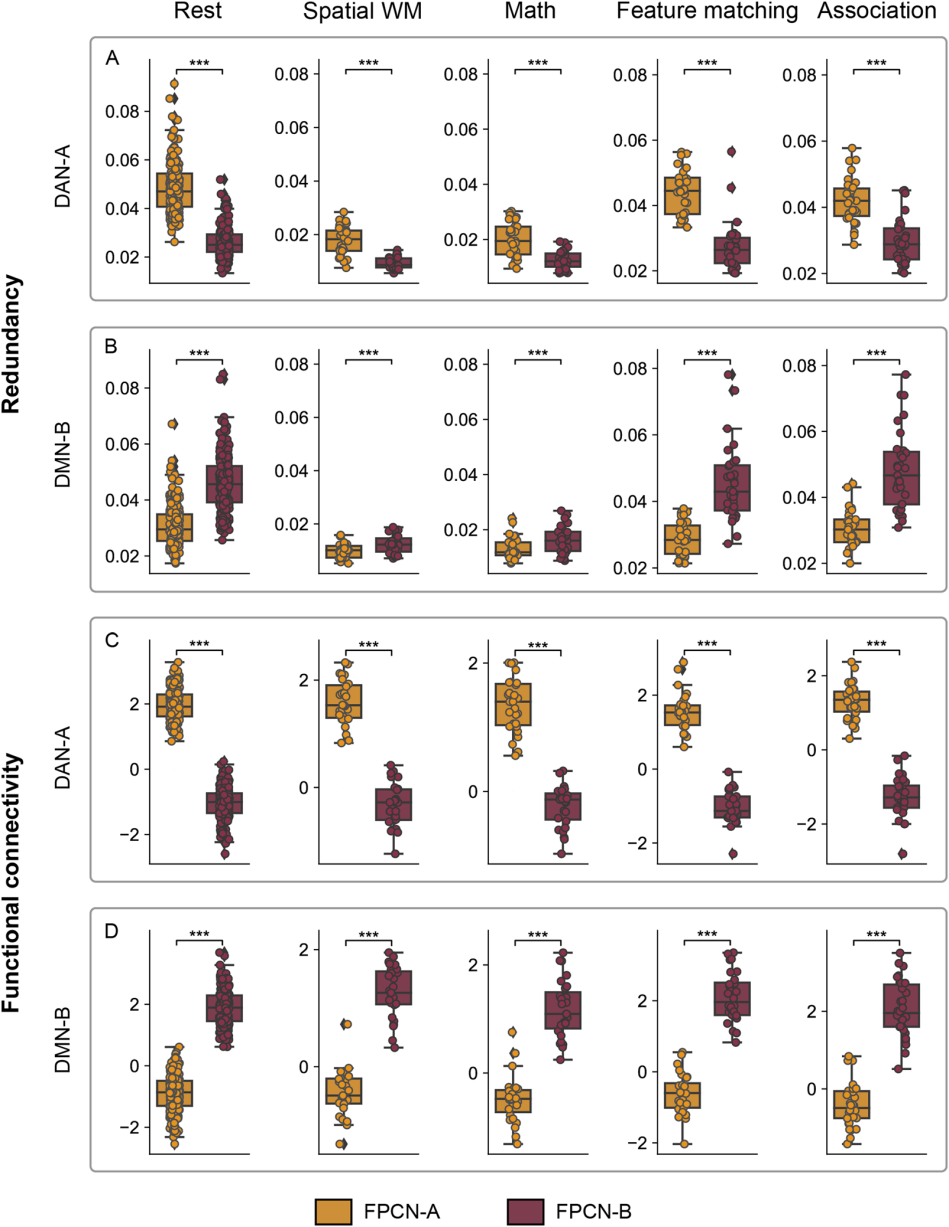
FPCN-A and FPCN-B interacted with different networks. FPCN-A consistently interacted with DAN-A while FPCN-B consistently interacted with DMN-B across different conditions. ***A***, ***C***, FPCN-A and DAN-A showed stronger redundancy and functional connectivity than FPCN-B and DAN-A. ***B***, ***D***, FPCN-B and DMN-B showed stronger redundancy and functional connectivity than FPCN-A and DMN-B. (***p = 0.001, FDR corrected).

To further characterize how topography affects functional interaction, we examined the following: (1) redundancy, which quantifies how much information about the brain's future trajectory is shared across brain regions, and (ii) functional connectivity between parcels, estimated by computing Pearson’s correlation coefficients between their time series. These measures revealed the same pattern as the network classification analysis: FPCN-A showed greater functional interaction with DAN-A and FPCN-B showed greater functional interaction with DMN-B. These patterns were observed during rest and all the tasks (*p* < 0.05, FDR corrected; Fig. 5, Tables 2 and 3). In summary, both redundancy and connectivity analyses indicated greater functional similarity between FPCN subnetworks and the networks they were closer to (DAN; DMN). In this way, the topography of FPCN subnetworks partly determines their functional tuning.

**Table 2.** DAN-A showed greater redundancy with FPCN-A than with FPCN-B; DMN-B showed the opposite pattern

Network	Task	Mean redundancy		FPCN-A vs FPCN-B		
		FPCN-A	FPCN-B	*t*	*p*	*q*
DAN-A	Rest	0.048	0.026	38.033	1.53 × 10^−104^	2.32 × 10^−75^
	Spatial WM	0.018	0.009	9.360	4.06 × 10^−10^	2.51 × 10^−7^
	Math	0.020	0.012	10.087	7.95 × 10^−11^	9.52 × 10^−10^
	Feature matching	0.044	0.027	11.408	1.95 × 10^−12^	3.64 × 10^−12^
	Association	0.042	0.030	9.544	1.34 × 10^−10^	2.67 × 10^−11^
DMN-B	Rest	0.031	0.046	−21.934	1.22 × 10^−59^	1.57 × 10^−20^
	Spatial WM	0.010	0.012	−5.010	2.70 × 10^−5^	1.85 × 10^−7^
	Math	0.013	0.016	−4.714	6.06 × 10^−5^	1.32 × 10^−7^
	Feature matching	0.029	0.045	−9.071	4.22 × 10^−10^	8.05 × 10^−5^
	Association	0.031	0.048	−9.519	1.42 × 10^−10^	6.75 × 10^−7^

*q* is the FDR-corrected *p* value.

**Table 3.** DAN-A showed greater functional connectivity (FC) with FPCN-A than with FPCN-B; DMN-B showed the opposite pattern

Network	Task	Mean FC		FPCN-A vs FPCN-B		
		FPCN-A	FPCN-B	*t*	*p*	*q*
DAN-A	Rest	1.955	−1.026	73.471	2.01 × 10^−168^	2.01 × 10^−167^
	Spatial WM	1.589	−0.326	17.545	6.24 × 10^−16^	8.79 × 10^−16^
	Math	1.341	−0.226	15.901	6.53 × 10^−15^	6.53 × 10^−15^
	Feature matching	1.545	−1.045	22.901	3.20 × 10^−19^	6.40 × 10^−19^
	Association	1.299	−1.262	22.413	7.33 × 10^−20^	1.99 × 10^−19^
DMN-B	Rest	−0.893	1.893	−64.832	8.23 × 10^−156^	4.12 × 10^−155^
	Spatial WM	−0.443	1.273	−17.458	7.03 × 10^−16^	8.79 × 10^−16^
	Math	−0.484	1.175	−17.281	8.98 × 10^−16^	9.98 × 10^−16^
	Feature matching	−0.610	2.100	−24.169	7.98 × 10^−20^	1.99 × 10^−19^
	Association	−0.419	2.059	−17.887	3.31 × 10^−17^	5.51 × 10^−17^

*q* is the FDR-corrected *p* value. The mean FC values are presented in adjusted *z* scores accounting for temporal autocorrelation (see Materials and Methods, Constructing fMRI functional connectivity matrices for detailed information).

#### FPCN-A regions showed greater activation in more demanding conditions, while FPCN-B regions showed the opposite pattern

Given that FPCN-A and FPCN-B occupy different topographical positions on the cortical surface and show different patterns of interaction with DAN-A and DMN-B, we might expect them to show different responses to control demands across tasks that differentially rely on DAN and DMN. To test this hypothesis, we examined univariate effects of task difficulty at the whole-brain level. In the working memory tasks, we contrasted hard and easy conditions, while in the long-term memory tasks, we designed experiments to examine parametric effects of difficulty (manipulations of feature similarity in the semantic feature matching task and association strength in the semantic association task). Detailed information on these manipulations can be found in Materials and Methods, Semantic feature matching task and Semantic association task. All *p* values were corrected using FDR at *p* < 0.05. For clarity, we focus on the results of the four networks of interest.

**Figure 6.**
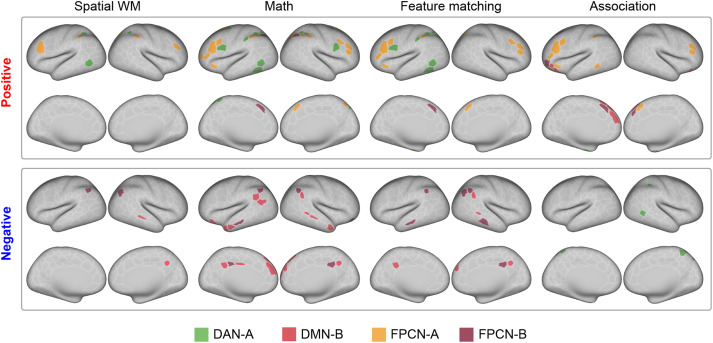
Modulation patterns of activation by task difficulty were similar for FPCN-A and DAN-A and FPCN-B and DMN-B. FPCN-A showed stronger activation in hard than easy trials across semantic and nonsemantic tasks. In contrast, FPCN-B typically deactivated in response to difficulty together with DMN-B. We examined univariate effects of task difficulty in the nonsemantic tasks by contrasting hard and easy conditions. We examined the difficulty effect in the long-term memory tasks by examining parametric effects of feature similarity in the feature matching task and association strength in the association task, respectively.

FPCN-A showed stronger activation in hard than that in easy trials across semantic and nonsemantic tasks (Fig. 6). DAN-A also showed positive effects of difficulty in the spatial working memory, math, and semantic feature matching tasks yet exhibited deactivation during more difficult decisions in the semantic association task. In this way, FPCN-A showed a consistent response to difficulty irrespective of the task context (as expected for regions of the “multiple demand network”; Duncan, 2010), while DAN-A was not always engaged by semantic difficulty. In contrast, FPCN-B and DMN-B showed little or no positive response to difficulty, although FPCN-B parcels in the dorsomedial prefrontal cortex did show a stronger response to more demanding decisions across math, feature matching and semantic association tasks. FPCN-B typically deactivated in response to difficulty, along with DMN-B (although this pattern was not observed for the semantic association task). These results highlight the need to explain how topography not only supports differentiation of function within FPCN subnetworks but also their flexible engagement in different task contexts.

### The interaction patterns of FPCN-A varied across tasks, while FPCN-B showed stable patterns

Our final analyses tested how the interaction patterns of FPCN subnetworks varied across tasks tapping working and long-term memory. FPCN-A was recruited by difficult decisions across all four tasks and was expected to show task-dependent interaction patterns, since domain-general control regions show highly flexible interaction patterns (Cole et al., 2013; Dixon et al., 2018). However, regions that support controlled retrieval from long-term memory are thought not contribute to control across domains (Wang et al., 2018; Gao et al., 2021; Chiou et al., 2022). Motivated by these findings, we asked whether FPCN-A would show greater interaction with DAN-A during nonsemantic working memory tasks and with FPCN-B in semantic tasks (examining network misclassification, redundancy, and functional connectivity). We then asked whether FPCN-B would show consistent interaction with FPCN-A and DMN-B across task contexts.

#### FPCN-A was more likely to be misclassified as DAN-A in the nonsemantic tasks but as FPCN-B in the semantic association task

We examined functional network similarity by analyzing the classification output (i.e., the confusion matrix) for each task, focusing on differences in the probability of misclassifying FPCN-A as DAN-A compared with FPCN-B across tasks. As expected, network similarity varied across tasks (Fig. 4*A*). FPCN-A was more likely to be misclassified as DAN-A than as FPCN-B in nonsemantic tasks (spatial working memory task: *t*_(27)_ = 3.913, *p* = 0.002, paired *t* test; math task: *t*_(27)_ = 5.294, *p* < 0.001, paired *t* test), but there was no significant difference between these networks in the feature matching task when participants needed to retrieve long-term memory according to external goals (*t*_(28)_ = 0.217; *p* = 0.83; paired *t* test). FPCN-A was more likely to be misclassified as FPCN-B than as DAN-A in the semantic association task (*t*_(30)_ = −3.00; *p* = 0.008; paired *t* test). All *p* values are corrected using FDR. In summary, FPCN-A varied its similarity to DAN-A and FPCN-B networks depending on the task state. This shows how misclassification effects go beyond patterns related to spatial adjacency to encompass task states. This shift in misclassification contradicts the typical assumption that networks and regions would always most closely resemble those that they are adjacent to on the cortical surface. While topography constrains the brain's coupling pattern, it does not determine it entirely.

#### FPCN-A showed greater functional interaction with DAN-A than with FPCN-B in the nonsemantic tasks and the opposite pattern in the semantic tasks

**Figure 7.**
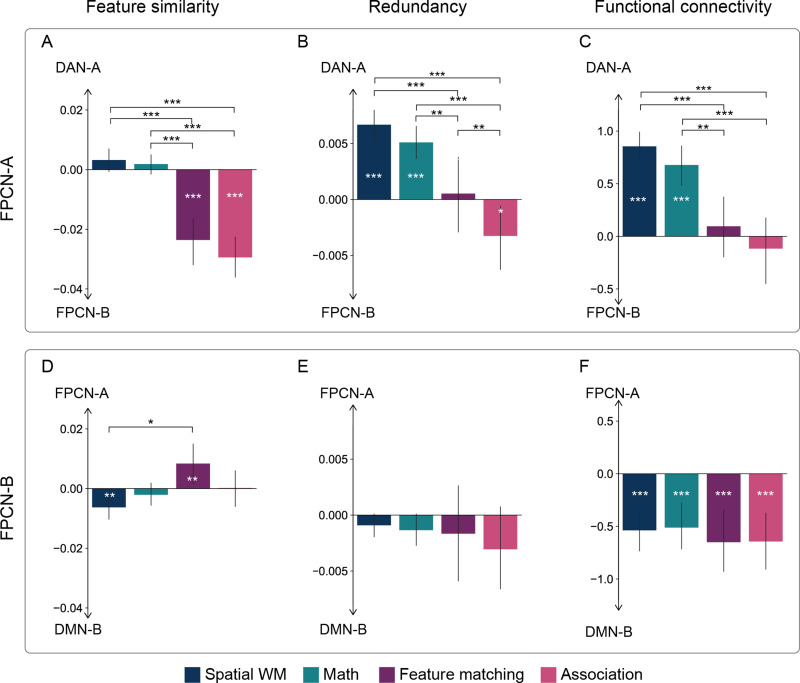
The interaction patterns of FPCN-A varied across tasks, while FPCN-B showed stable patterns. The values in the top panel represent the interaction difference between FPCN-A and DAN-A versus FPCN-A and FPCN-B: positive values mean FPCN-A showed greater interaction with DAN-A than with FPCN-B. The values in the bottom panel represent the interaction difference between FPCN-B and FPCN-A versus FPCN-B and DMN-B: positive values mean FPCN-B showed greater interaction with FPCN-A than with DMN-B. ***A–C***, The interaction differences in feature similarity, redundancy, and functional connectivity between FPCN-A and DAN-A versus FPCN-A and FPCN-B were greater in the nonsemantic working memory tasks than in the semantic tasks. The task differences reflected greater interaction of FPCN-A with DAN-A than with FPCN-B in the nonsemantic working memory tasks but the opposite pattern during the retrieval of knowledge from long-term memory. ***D–F***, There were no interaction differences in redundancy and functional connectivity between FPCN-B and FPCN-A versus FPCN-B and DMN-B across tasks. The difference in feature similarity was greater in the semantic feature matching task than in the spatial working memory task because FPCN-B showed greater interaction with FPCN-A than with DMN-B in the semantic feature matching task but showed the opposite pattern in the spatial working memory task. The white asterisk means the interaction difference was significant for that task at a specific threshold (*p = 0.05; **p = 0.01; ***p = 0.001 by permutation based maximum *t* tests, FWE corrected). The black asterisk means the interaction difference was significant across task pairs at a specific threshold (*p = 0.05; **p = 0.01; ***p = 0.001 by permutation based maximum *t* tests, FWE corrected).

Next, we considered differences in feature similarity, redundancy, and functional connectivity between tasks, examining interaction of FPCN-A with DAN-A compared with FPCN-B. FPCN-A's interaction to DAN-A compared with FPCN-B was consistently greater for the nonsemantic than for the semantic tasks (*p* < 0.001; FWE corrected; Fig. 7). FPCN-A showed greater feature similarity, shared more information, and had stronger functional connectivity with DAN-A in the spatial working memory and math tasks but interacted more with FPCN-B in the semantic tasks involving long-term memory (*p* < 0.001; FWE corrected; Fig. 7). This shift in functional interaction again challenges the prevailing assumption that networks and regions would consistently resemble their most adjacent cortical neighbors.

#### FPCN-B showed consistent interaction with FPCN-A and DMN-B across tasks

Having examined the flexible interaction patterns of FPCN-A, we asked if FPCN-B showed similar effects. We compared the probability that FPCN-B was misclassified as FPCN-A or as DMN-B in each task, as these were the most confusable networks for FPCN-B. As shown in Figure 4*B*, misclassification was greater for FPCN-A than that for DMN-B in the semantic feature matching task, when the participants needed to retrieve long-term memory according to external goals (*t*_(28)_ = 2.470; *p* = 0.019; paired *t* test). FPCN-B was misclassified as FPCN-A and DMN-B equally often in the semantic association task (*t*_(30)_ = 1.811; *p* = 0.080; paired *t* test) and in the nonsemantic tasks (spatial working memory task: *t*_(27)_ = −1.037, *p* = 0.309, paired *t* test; math task: *t*_(27)_ = −1.326, *p* = 0.245, paired *t* test). All *p* values are corrected using FDR. These results show that although FPCN-B can resemble FPCN-A when people engage in controlled retrieval from long-term memory, this network also has a strong similarity with DMN-B across task contexts.

Finally, we quantified differences in feature similarity, redundancy, and functional connectivity for FPCN-B across tasks, examining interaction of this network with DMN-B and FPCN-A. In contrast to FPCN-A's flexibility, FPCN-B showed a relatively stable interaction pattern. There were no significant differences in redundancy or functional connectivity across tasks (Fig. 7*E*,*F*; *p* > 0.05, uncorrected). FPCN-B showed equal redundancy with FPCN-A and DMN-B at rest and in all the tasks, with no significant differences between tasks (Fig. 7*E*; *p* > 0.05, uncorrected). FPCN-B always showed greater functional connectivity with DMN-B than with FPCN-A (Fig. 7*F*; *p* < 0.05, FWE corrected). FPCN-B showed greater feature similarity with FPCN-A than with DMN-B in the semantic feature matching task (*t*_(28)_ = 2.317; *p* = 0.033; paired *t* test) but showed the opposite pattern in the spatial working memory task (*t*_(27)_ = −3.153; *p* = 0.005; paired *t* test) and this task difference was significant (Fig. 7*D*; *p* < 0.002, FWE corrected). FPCN-B more closely resembled FPCN-A when people engaged in controlled retrieval from long-term memory, but overall FPCN-B showed relatively consistent network interaction across tasks.

## Discussion

**Figure 8.**
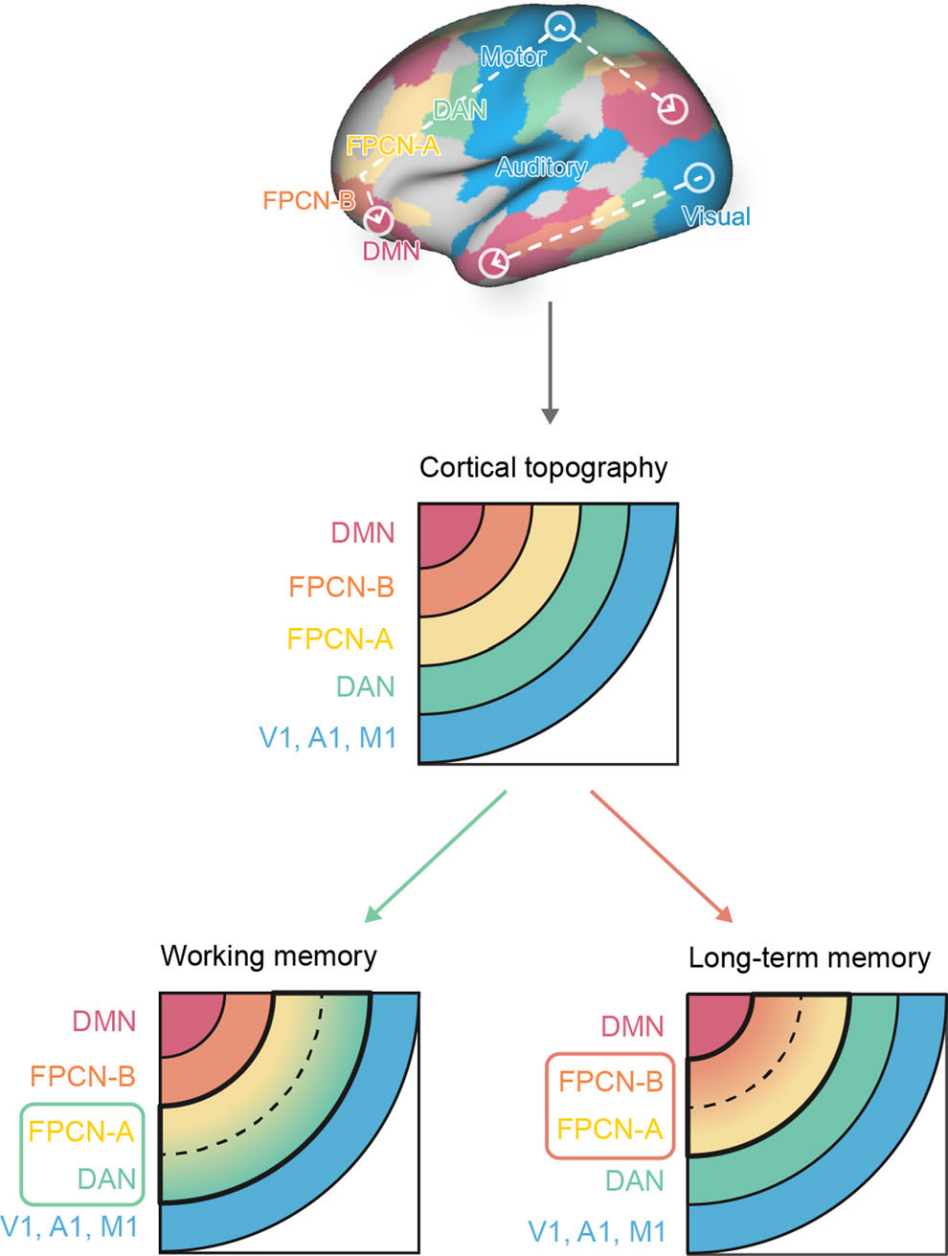
A schematic of topographical relationships of networks and how this allows them to change their interaction patterns according to task demands to support flexible behavior, after Mesulam (1998). FPCN-A and FPCN-B are situated between DAN and DMN, and they are influenced by their adjacent networks, allowing them to resolve competition and keep a functional balance between goal-oriented attentional mechanisms supporting working memory (drawing on DAN) and retrieval from long-term memory (supported by DMN). The topographical location of FPCN-A—situated between DAN and FPCN-B—allows it to shift its interaction toward attentional systems or memory systems according to task demands. In a changing environment, the engagement of externally oriented attentional mechanisms fractionates regions implicated in cognitive control, such that FPCN-A interacts more with DAN-A than with FPCN-B during working memory tasks. This architecture might prevent interference from memory-based schema. When cognition instead needs to be guided by long-term memory, FPCN-A interacts with FPCN-B, making this network less able to track fluctuating environmental changes but allowing it to integrate information from DMN.

Our study shows that cortical topography, in conjunction with the underlying anatomy, provides a landscape in which flexible changes in neural function across situations can be understood. This landscape offers an architecture that supports the flexible deployment of distinct cognitive modes. By this account, FPCN subnetworks supporting distinct aspects of cognitive control are topographically positioned and anatomically similar to their adjacent systems, allowing them to interact in a context-specific manner (Fig. 8). FPCN-A and FPCN-B are proximal to DAN and DMN, respectively, and this proximity is reflected by key anatomical features, including myelin content, the degree of cortical expansion, and cross-species similarity. This anatomical similarity complements the topographical organization of FPCN into adjacent subsystems, and these aspects together account for important features of FPCN's functional behavior. FPCN-A is more interacted with attention regions (particularly DAN-A), while FPCN-B is more interacted with memory regions (particularly DMN-B). Despite differences in interaction with DAN-A and DMN-B, these relationships can change across contexts. FPCN-A shows stronger functional interaction to DAN-A during working memory tasks and to FPCN-B during tasks more reliant on long-term memory. This shift in interaction challenges the prevailing assumption that networks and regions would consistently resemble their most adjacent cortical neighbors. These findings align with the expectation that the FPCN functions as a unified system, particularly when long-term knowledge supports behavior. However, it segregates into discrete subsystems with different interaction patterns when long-term memory is less supportive, under which circumstances it no longer behaves as a coherent unit. FPCN-B shows more deactivation in response to demanding tasks and maintains a pattern of interaction with both FPCN-A and DMN-B across tasks. The relative flexibility in FPCN-A and the relative stability of FPCN-B explains how people can utilize context-specific working memory and long-term memory to support flexible behavior.

These findings confirm that FPCN-A is closer to sensory-motor regions, positioned between DAN and FPCN-B on the cortical hierarchy, and can change its interaction with these networks depending on the task demands. In contrast, FPCN-B lies between FPCN-A and DMN-B and interacts with both networks (Fig. 8). In this way, the topographical organization of cognitive control regions allows FPCN-A to show the characteristics of a highly flexible “multiple-demand” network, supporting executive control processes across domains (Duncan, 2010; Woolgar et al., 2011, 2016, 2018; Fedorenko et al., 2013; Jackson and Woolgar, 2018; Assem et al., 2020, 2022, 2023), while the neighboring FPCN-B shows the characteristics of a memory control network (Jefferies, 2013; Noonan et al., 2013; Gao et al., 2021; Hodgson et al., 2022). This architecture might allow FPCN to be influenced by both DAN and DMN at different times, even though these networks are sometimes anticorrelated (Anderson et al., 2011; Davey et al., 2016; Chen et al., 2017; Dixon et al., 2017; Craig et al., 2018; Wang et al., 2021; Chiou et al., 2022). In this way, our findings explain how previous studies found both functional dissociations and similarities between FPCN and DMN across different task contexts (Dixon et al., 2018; González-García et al., 2018; Wang et al., 2018, 2021).

The ability of FPCN-A to change its activity in a context-specific manner indicates that this network might serve as a “dynamic core” controlling information flow by modulating network interactions in a context-sensitive fashion (Sliwinska et al., 2017; Shine et al., 2019). Dynamic core regions are involved in multiple tasks, can integrate more specialized brain regions, and alter their baseline communication dynamics to support task-specific computations (Koch et al., 2016; Herrmann and Johnsrude, 2018; Khambhati et al., 2018; Finc et al., 2020; Ritz et al., 2022; Turnbull et al., 2019). In a changing environment, the interaction of FPCN-A with externally oriented attentional mechanisms appears to break apart the FPCN, such that FPCN-A interacts more with DAN-A than with FPCN-B (Boag et al., 2021). This architecture might prevent interference from memory-based schema that conflict with the veridical state of the environment. When cognition instead needs to be guided by long-term memory, FPCN-A interacts more with FPCN-B, making this network less able to track fluctuating environmental changes but allowing it to integrate information from DMN.

Our findings are broadly consistent with the tethering hypothesis (Buckner and Krienen, 2013), which posits that proximity to sensory-motor regions constrains brain function. By extension, this account suggests that the relative separation of FPCN-A from DMN helps protect information in working memory from biases influenced by conceptual knowledge, emotion, and motivations. Conversely, the proximity of FPCN-B to DMN enables it to support the retrieval of abstract and heteromodal concepts and to reflect relevant information in memory even when this is at odds with the external environment (Murphy et al., 2018; Hawkins et al., 2022). In tasks requiring controlled memory retrieval, these two control systems are expected to work together, allowing the representation of goals in working memory to interact with abstract semantic representations (Davey et al., 2016; Wang et al., 2021; Chiou et al., 2022; Lyu et al., 2022; Ovando-Tellez et al., 2022b, a; Gasser and Davachi, 2023; Halpern et al., 2023). Importantly, our study suggests that part of this flexibility may be reflected in the accompanying anatomical differences that mirror the topography of these networks.

There are remaining questions to be addressed. First, we have yet to establish whether the functional architecture we describe is relevant to all aspects of long-term memory, as our data are restricted to verbal semantic judgements. Future research should examine nonverbal semantic tasks and aspects of long-term memory beyond the semantic domain. Similarly, this architecture for flexible cognitive control may not extend to all working memory tasks or to controlled perceptual-motor tasks. Multiple cognitive dimensions might influence network interactions in similar ways. We found that two working memory tasks, involving spatial working memory and math, showed largely consistent patterns despite their difference in representational content (concrete perceptual vs abstract). Similarly, our two long-term memory tasks, despite the distinction between perceptual and abstract content, displayed similar interaction patterns. Nevertheless, our task comparisons affect both working/long-term memory demands and representational content simultaneously, as we do not compare short-term and long-term memory versions of tasks that involve the same information. Previous studies have shown that, in addition to their role in working memory, DAN and FPCN-A can support long-term memory when it involves retrieving perceptual features of situations (Stawarczyk et al., 2018); moreover, DMN and FPCN-B support long-term memory but can be involved in working memory when social or abstract information is held in mind (Meyer et al., 2012). Although this study observed different patterns of network interaction for long-term and working memory tasks, similar distinctions in network interaction may arise from other types of task contrasts, such as those comparing more abstract versus more perceptual features in long-term memory. Other network interactions may also be important for flexible human cognition across a broader range of tasks, including the FPCN-C subnetwork, which was not found to strongly contribute to the tasks in this study (see additional analyses reported in Wang et al., 2023).

Future studies can directly compare the flexibility of FPCN-A and FPCN-B using the same participants and the same parcellation method. In this study, we adopted the individual-specific parcellation method from Kong et al. (2021), differing slightly from the approaches used in previous studies such as Dixon et al. (2018) and Murphy et al. (2020). This variation in parcellation approaches presents a challenge in directly comparing our results with these previous studies. It highlights the need to re-examine our findings using diverse parcellation strategies. In the future, it would be interesting to explore network interactions through the lens of synergy for a more comprehensive understanding of the neurocognitive processes underpinning mental flexibility, since synergy is thought to index integrative processes (Luppi et al., 2022, 2024; Varley et al., 2023).

Another unresolved question is whether similar topographically organized processes support functional flexibility in a comparable way for other networks. Future studies can explore subnetworks of DAN or DMN to establish if the patterns described here are unique to FPCN or reflect more general principles. Research should also assess the extent to which the mechanisms for flexible control described here relate to individual differences in cognitive performance and cognition in daily life, particularly whether topographical differences predict these individual differences in behavior. Finally, while it is extremely challenging to examine the causal relationship between topography and function, computational simulations and studies of people with sensory impairment may help to elucidate this link (Schone et al., 2021; Kikkert et al., 2024).

In conclusion, this study demonstrates how brain organization supports mental flexibility. We establish that flexible human behavior is partly supported by the topographical separation of the FPCN into subnetworks that are proximal to DAN and DMN. This topographical organization enables distinct patterns of interaction to emerge in tasks underpinned by working memory and long-term knowledge, explaining how the brain maintains a functional balance between states that rely on top-down attention to the external environment and those that require retrieval of information from long-term memory.
